# Computational Modeling of Information Propagation during the Sleep–Waking Cycle

**DOI:** 10.3390/biology10100945

**Published:** 2021-09-22

**Authors:** Farhad Razi, Rubén Moreno-Bote, Belén Sancristóbal

**Affiliations:** 1 Barcelona School of Design and Engineering, Universitat de Vic—Universitat Central de Catalunya, 08002 Barcelona, Spain; frazi@elisava.net; 2 Center for Brain and Cognition, Department of Information and Communications Technologies, Universitat Pompeu Fabra, 08018 Barcelona, Spain; ruben.moreno@upf.edu

**Keywords:** synaptic homeostasis hypothesis, information propagation, cortical effective connectivity, neural-mass model, wakefulness, NREM sleep, local field potential

## Abstract

**Simple Summary:**

During the deep phases of sleep we do not normally wake up by a thunder, but we nevertheless notice it when awake. The exact same sound gets to our ears and cortex through the thalamus and still, it triggers two very different responses. There is growing experimental evidence that these two states of the brain—sleep and wakefulness—distribute sensory information in different ways across the cortex. In particular, during sleep, neuronal responses remain local and do not spread out across distant synaptically connected regions. On the contrary, during wakefulness, stimuli are able to elicit a wider spatial response. We have used a computational model of coupled cortical columns to study how these two propagation modes arise. Moreover, the transition from sleep-like to waking-like dynamics occurs in agreement with the synaptic homeostasis hypothesis and only requires the increase of excitatory conductances. We have found that, in order to reproduce the aforementioned observations, this parameter change has to be selectively applied: synaptic conductances between distinct columns have to be potentiated over local ones.

**Abstract:**

Non-threatening familiar sounds can go unnoticed during sleep despite the fact that they enter our brain by exciting the auditory nerves. Extracellular cortical recordings in the primary auditory cortex of rodents show that an increase in firing rate in response to pure tones during deep phases of sleep is comparable to those evoked during wakefulness. This result challenges the hypothesis that during sleep cortical responses are weakened through thalamic gating. An alternative explanation comes from the observation that the spatiotemporal spread of the evoked activity by transcranial magnetic stimulation in humans is reduced during non-rapid eye movement (NREM) sleep as compared to the wider propagation to other cortical regions during wakefulness. Thus, cortical responses during NREM sleep remain local and the stimulus only reaches nearby neuronal populations. We aim at understanding how this behavior emerges in the brain as it spontaneously shifts between NREM sleep and wakefulness. To do so, we have used a computational neural-mass model to reproduce the dynamics of the sensory auditory cortex and corresponding local field potentials in these two brain states. Following the synaptic homeostasis hypothesis, an increase in a single parameter, namely the excitatory conductance ${\bar{g}}_{\mathrm{AMPA}}$, allows us to place the model from NREM sleep into wakefulness. In agreement with the experimental results, the endogenous dynamics during NREM sleep produces a comparable, even higher, response to excitatory inputs to the ones during wakefulness. We have extended the model to two bidirectionally connected cortical columns and have quantified the propagation of an excitatory input as a function of their coupling. We have found that the general increase in all conductances of the cortical excitatory synapses that drive the system from NREM sleep to wakefulness does not boost the effective connectivity between cortical columns. Instead, it is the inter-/intra-conductance ratio of cortical excitatory synapses that should raise to facilitate information propagation across the brain.

## 1. Introduction

Sensory information processing is not fully blocked during sleep since animals need to identify relevant stimuli for their survival. However, when compared to wakefulness, focused attention to the environment is lost while we sleep. Such reduced sensitivity in the detection of external signals leads to a decrease in sensory awareness. Still, sleep is a vital recurrent state that reduces brain energy demands by slowing down the metabolic rate [1]. Behaviorally, this is clearly observed in the cessation of actions since sleep holds back reproduction, exploration, protection and nurture of the offspring. Electrophysiologically, the deep phases of sleep (NREM sleep) are distinguished from wakefulness based on the electrical activity emerging from neuronal populations. For instance, extracellular cortical recordings obtained during wakefulness show low-amplitude–high-frequency voltage fluctuations whereas during NREM sleep, and also during deep anesthesia [2,3,4], these fluctuations exhibit high-amplitude–low-frequency components and are bimodal [3,4,5,6,7,8]. The bimodality appears due to the alternation between periods of persistent firing activity (Up states) and silent periods (Down states), which provides the idiosyncratic dynamical features of NREM sleep, also known as slow wave activity (SWA). This leads to a reduction of the overall firing rate of both excitatory and inhibitory neuronal cell types in the time course of NREM sleep [4,5,6,7,8].

Despite these differences, neurons in the primary auditory cortex show comparable responses during sleep and wakefulness when pure tones are delivered to rats [7] and primates [9]. These experimental results challenge the hypothesis that during sleep cortical responses are weakened through thalamic gating, i.e., the mechanism by which thalamic spindles could prevent a reliable transmission of external signals to sensory cortices during sleep [10]. Interestingly, in higher-order cortical areas during sleep—in the perirhinal cortex in rats [8] and the human cingulate and prefrontal cortex [11] and during anesthesia—in the association cortex in humans [12], auditory responses are attenuated. These studies imply that information gating must occur within brain structures higher up the stream of sensory processing and seem to be more consistent with a breakdown of functional cortical connectivity rather than of thalamocortical connectivity. Indeed, fMRI data collected in humans indicate a reduced global effective connectivity during NREM sleep [13]. Even in the presence of cycling alternating patterns (CAP), a sign of sleep instability, EEG-based functional connectivity shows a small-world network type of structure [14]. Moreover, cortical responses to transcranial magnetic stimulation (TMS) have been shown to be local during NREM sleep and do not propagate to connected brain regions [15,16].

One possible neural correlate of decreased sensory responsiveness in the non-primary cortex during NREM sleep could be the downscaling of the conductance of excitatory synapses, a hypothesis known as synaptic homeostasis hypothesis (SHY) [17,18,19,20,21]. Experimental evidence [22,23,24,25] shows that potentiation of synapses during wakefulness caused by synaptic plasticity is compensated during NREM sleep with a general reduction of synaptic strength. We implement this synaptic conductance downscaling in our computational study by tuning a single parameter that is biophysically equivalent to an excitatory synaptic conductance. Moreover, decreasing its value shifts the model from wakefulness to NREM sleep. To our knowledge, this is the first mathematical model, among all the different proposals [26,27,28,29,30], that reconciles these two important findings of sleep research: synaptic downscaling and the simultaneous emergence of slow rhythms as the brain enters the deep phases of sleep from wakefulness.

We aim at understanding, within the neural mass model formalism used in this study and others [27,31], whether SHY not only explains the aforementioned change in brain dynamics, but also in the propagation span of evoked activities occurring throughout the sleep–waking cycle (SWC). To do so, we have simulated the local field potential (LFP) signals of two cortical columns operating in either NREM sleep or wakefulness. One cortical column—the perturbed one—responds to an externally applied excitatory input, reproducing the activation of a primary auditory area by a sound or a superficial region of the cerebral cortex stimulated by TMS. A second cortical column—the unperturbed one—is bidirectionally coupled to the perturbed column and does not directly receive the external input, as occurs with non-primary auditory areas and neighboring regions. We quantify the propagation of this external input as a function of the strength of the inter-column connectivity in each brain state. We show that the upscaling of the conductance of excitatory synapses shifts the LFP dynamics from NREM sleep to wakefulness in both columns, as they are intrinsically identical. However, a general build-up of this conductance does not lead to the propagation of the external input from the perturbed cortical column to the unperturbed cortical column. Interestingly, we have found that the unperturbed cortical column responds only significantly when the increase in the excitatory conductance selectively takes place in the inter-column synapses with respect to the intra-column connectivity. This finding points to a novel and different interpretation of SHY, namely that the tuning of synaptic conductances could be distance-dependent.

Our results are presented as follows. First, we introduce the model for a single cortical column in order to validate the parameter space where the system exhibits NREM and wakefulness dynamics. Second, we extend the network to two cortical columns coupled bidirectionally with excitatory synapses to generalize the results to a more complex connectivity diagram. Note that now the change in synaptic conductance that allows the system to shift dynamics, also affects the inter-column connectivity and thus directly influences signal transmission between columns. Finally, we show how efficient input propagation observed during wakefulness, and reduced during NREM sleep, can be obtained.

## 2. Materials and Methods

In the following section, we first describe the neural mass formalism [27,31] that is able to reproduce the extracellular dynamics of a single cortical column. Increasing the strength of the intra-cortical excitatory synapses, in agreement with SHY, allows us to move from NREM-like activity to wakefulness. Finally, we expand the model to two mutually connected cortical columns.

### 2.1. Neural Mass Model

**Figure 1:**
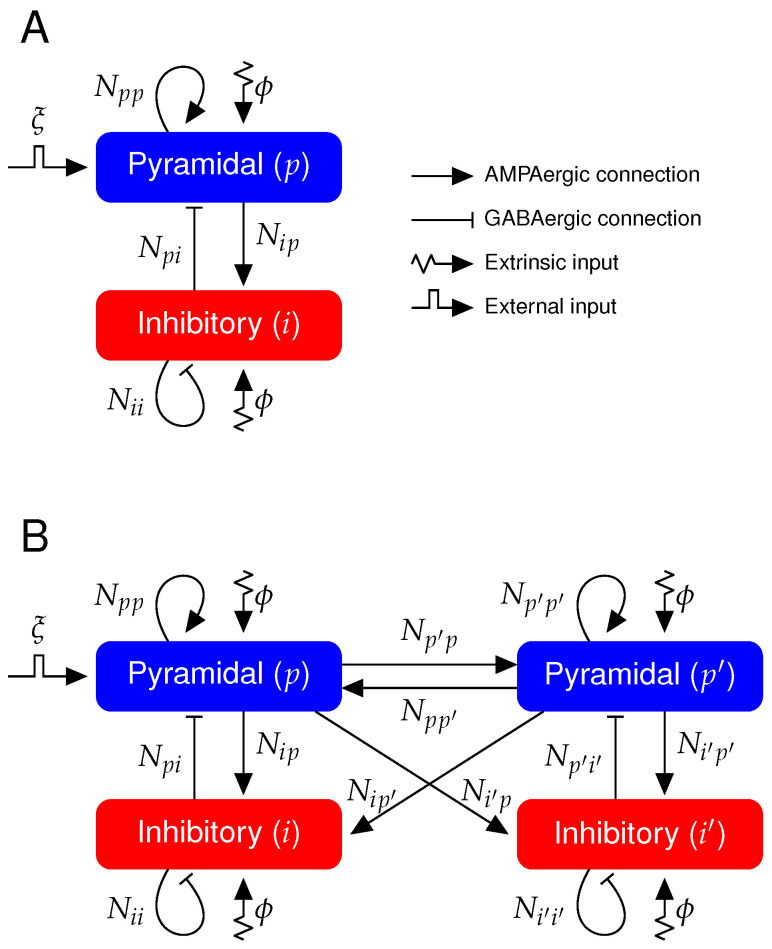
Diagram of the neural mass model. (**A**) One cortical column containing one pyramidal and one inhibitory population. Coupling between pyramidal and inhibitory populations are mediated through AMPAergic and GABAergic connections (see Table 1 and Table 2 for symbol description and parameter values). (**B**) Neural mass model describing two mutually coupled cortical columns. Connections between two cortical columns are only AMPAergic, i.e., only the pyramidal populations target both pyramidal and inhibitory populations from the other cortical column (see Table 1 and Table 3 for symbol description and parameter values). ξ represents the transient external input impinging only in one cortical column.

Recordings of the extracellular neuronal field capture the superposition of all transmembrane currents, such as the synaptic and ionic currents [32]. In order to reproduce the dynamical features of these signals, we use a neural mass model [27,31] representing the average membrane potential of a neural cortical column that leads to the LFP characteristics of the SWC. A single cortical column consists of pyramidal, *p*, and inhibitory, *i*, population mutually connected (see Figure 1A for schematic).

The dynamics of the average membrane potential, $V_{p/i}$, is governed by the mathematical formalism of the classical conductance-based model [33] with one leak, two synaptic currents and one activity-dependent potassium current as follows:

(1) $$\begin{array}{cc} \tau_{p}{\dot{V}}_{p} & =-I_{L}^{p}-I_{\mathrm{AMPA}}^{p}-I_{\mathrm{GABA}}^{p}-\tau_{p}C_{m}^{-1}I_{\mathrm{KNa}}, \end{array}$$

(2) $$\begin{array}{cc} \tau_{i}\dot{V_{i}} & =-I_{L}^{i}-I_{\mathrm{AMPA}}^{i}-I_{\mathrm{GABA}}^{i}, \end{array}$$

where $\tau_{p/i}$, $I_{L}^{p/i}$, $I_{AMPA}^{p/i}$ and $I_{GABA}^{p/i}$ are, respectively, the membrane time constant and the leak, AMPAergic and GABAergic current of population *p* or *i*. $I_{\mathrm{KNa}}$ is the activity-dependent potassium current of the pyramidal population (see Section 2.3). $C_{m}$ is the membrane capacitance in the Hodgkin–Huxley model.

The average firing rate of each population is modeled as an instantaneous function of the average membrane potential $V_{p/i}$ according to a sigmoid function [34,35]:

(3) $$Q_{p/i}\left(V_{p/i}\right)=Q_{p/i}^{max}\frac{\left(1+\tanh\left(C\left(V_{p/i}-\theta_{p/i}\right)/\sigma_{p/i}\right)\right)}{2},$$

where $Q_{p/i}^{max}$, $\theta_{p/i}$ and $\sigma_{p/i}$ represent the maximal firing rate, firing rate threshold and inverse neural gain of population *p* or *i*, respectively (see Table 1 and Table 2 for symbol description and parameter value, respectively). Here, $C=\frac{\pi }{2\sqrt{3}}$ is a constant linking the neural gain to the slope of the sigmoid function [27].

### 2.2. Model Synapses

The presynaptic activities have an intrinsic and an extrinsic origin. The intrinsic or recurrent activity is the input each population receives from local pyramidal and inhibitory populations. The extrinsic input comes from non-explicitly modeled distant cortical columns that impinges on population *p* and *i* only through excitatory synapses. This is in agreement with the morphology of cortical neurons, whereby pyramidal neurons have larger projecting axons with respect to the more local and constrained range of inhibitory axons [36,37,38]. This has also been reflected in numerous computational studies [30,39,40,41,42].

Given a presynaptic population $k^{\prime }$ and a postsynaptic population *k*, the time course of synaptic activity, $s_{kk^{\prime }}$, at time *t*, depends on the pyramidal or inhibitory nature of the presynaptic population $k^{\prime }$. Synapses are considered AMPAergic for excitation, i.e., when population $k^{\prime }$ is the pyramidal one, and GABAergic for inhibition, i.e., when population $k^{\prime }$ is the inhibitory one. The mathematical formalism adopted is the convolution of the presynaptic firing, $Q_{k^{\prime }}$, with the average synaptic response to a single spike $\alpha_{k^{\prime }}$ that has an exponential decay time course [43]:

(4) $$\begin{array}{cc} s_{kk^{\prime }} & =N_{kk^{\prime }}Q_{k^{\prime }}\left(V_{k^{\prime }}\right)\;⊛\;\alpha_{k^{\prime }}, \end{array}$$

(5) $$\begin{array}{cc} \alpha_{k^{\prime }} & =\gamma_{k^{\prime }}^{2}\;t\;\exp\left(-\gamma_{k^{\prime }}t\right), \end{array}$$

where $N_{kk^{\prime }}$ is the mean number of synaptic connections from presynaptic population $k^{\prime }$ to postsynaptic population *k*. $\gamma_{k^{\prime }}$ describes the time constant of the dynamics of a synapse activated by the presynaptic firing of population $k^{\prime }$. The set of Equations (4) and (5) lead to the second-order differential equation (derived in detail in Appendix A.1):

(6) $${\ddot{s}}_{kk^{\prime }}=\gamma_{k^{\prime }}^{2}\;\left(N_{kk^{\prime }}\;Q_{k^{\prime }}\left(V_{k^{\prime }}\right)+\phi -s_{kk^{\prime }}\right)\;-2\gamma_{k^{\prime }}\;{\dot{s}}_{kk^{\prime }},$$

where Equation (6) describes the AMPAergic and GABAergic synaptic activity due to the firing of the pyramidal and inhibitory population $k^{\prime }$, respectively, onto postsynaptic population *k*. Moreover, the noise ϕ is simulated independently for each cortical population as a Gaussian process with zero autocorrelation time constant and zero mean. We assume that the standard deviation of ϕ is greater during NREM, i.e., 1.8 ${\mathrm{ms}}^{-1}$, than during wakefulness, i.e., 1 ${\mathrm{ms}}^{-1}$, because in the former dynamics, the firing rate fluctuates largely between the Up and Down states. The AMPAergic and GABAergic synaptic currents are defined as:

(7) $$\begin{array}{cc} I_{\mathrm{AMPA}}^{k} & ={\bar{g}}_{\mathrm{AMPA}}^{\;k}\;s_{kp}\;\left(V_{k}-E_{\mathrm{AMPA}}\right), \end{array}$$

(8) $$\begin{array}{cc} I_{\mathrm{GABA}}^{k} & ={\bar{g}}_{\mathrm{GABA}}^{\;k}\;s_{ki}\;\left(V_{k}-E_{\mathrm{GABA}}\right), \end{array}$$

where ${\bar{g}}_{\mathrm{AMPA}}^{\;k}$ and ${\bar{g}}_{\mathrm{GABA}}^{\;k}$ are, respectively, the average AMPAergic and GABAergic conductance on population *k* (also referred to as the recurrent or intra-conductances). $E_{\mathrm{AMPA}}$ and $E_{\mathrm{GABA}}$ are the reversal potential of AMPAergic and GABAergic currents.

### 2.3. Activity-Dependent Potassium Current

SWA is the hallmark of neuronal cortical activity during NREM sleep and anesthesia. It consists of silent (Down) and persistent (Up) firing patterns of activity that alternate. Activation of the slow activity-dependent $K^{+}$ current has been suggested as one possible mechanism triggering Down state initiation [3,44,45,46]. The sodium-dependent potassium current $I_{\mathrm{KNa}}$ and sodium concentration [Na] are implemented in the model as:

(9) $$\begin{array}{cc} I_{\mathrm{KNa}} & ={\bar{g}}_{\mathrm{KNa}}\;\frac{0.37}{1+{\left(\frac{38.7}{\left[\mathrm{Na}\right]}\right)}^{3.5}}\;\left(V_{p}-E_{K}\right), \end{array}$$

(10) $$\begin{array}{cc} \tau_{\mathrm{Na}}\;\dot{\left[\mathrm{Na}\right]} & =\alpha_{\mathrm{Na}}Q_{p}\left(V_{p}\right)-{\mathrm{Na}}_{\mathrm{pump}}\left(\left[\mathrm{Na}\right]\right), \end{array}$$

where ${\bar{g}}_{\mathrm{KNa}}$, $E_{K}$, $\tau_{\mathrm{Na}}$ and $\alpha_{\mathrm{Na}}$ correspond to the average conductance of $I_{\mathrm{KNa}}$, the Nernst reversal potential of the $I_{\mathrm{KNa}}$ current, the time constant of extrusion of sodium concentration and the average influx of sodium concentration per firing. ${\mathrm{Na}}_{\mathrm{pump}}$ is a function representing sodium pumps that determines the extrusion of sodium concentration through sodium pumps (see Appendix A for details).

### 2.4. Upscaling the AMPAergic Conductance

The neural mass model proposed by [27,31] is able to generate the physiological characteristics of the sleeping cortex for particular values of the parameters. There, the authors showed that by reducing $\sigma_{p}$ and ${\bar{g}}_{\mathrm{KNa}}$, the model generates the low-amplitude–high-frequency fluctuations in the average membrane potential characteristic of wakefulness. However, with their proposed set of parameter values, the firing rate of the pyramidal population is saturated at its maximum, given by $Q_{p}^{\max}$ of the sigmoid function $Q_{p}\left(V_{p}\right)$. Therefore, we propose a different set of bifurcation parameters to place the model within the wakefulness regime.

We have chosen to upscale the conductance of excitatory synapses by increasing the average AMPAergic conductance, ${\bar{g}}_{\mathrm{AMPA}}^{\;k}$, to go from NREM sleep to wakefulness, which is in agreement with SHY. The average GABAergic conductance, ${\bar{g}}_{\mathrm{GABA}}^{\;k}$, has been modified on both pyramidal and inhibitory populations in order to maintain the steady state value of the average membrane potential of pyramidal $V_{p}$ and inhibitory $V_{i}$ populations equal to their values in the Up state during NREM sleep (see Table 2 to compare parameter values between brain states). These $V_{p}$ and $V_{i}$ values are determined by simulating 500 trials (10 s long in duration) of NREM dynamics. Up and Down events are determined from the LFP signals (see Section 2.8.1 below for a definition of LFP and Section 2.8.2 for the Up and Down separation method). Then, the peak of the distributions of $V_{p}$ and $V_{i}$ during the Up state events is used as the steady state value of the average membrane potential of pyramidal and inhibitory populations during wakefulness. The average GABAergic conductance on pyramidal and inhibitory populations is increased until these $V_{p}$ and $V_{i}$ values are obtained for the ${\bar{g}}_{\mathrm{AMPA}}^{\;k}$ values responsible of wakefulness (see Appendix B.1 for dynamical constraints on the average GABAergic conductance on pyramidal and inhibitory populations in the one-cortical-column scenario).

### 2.5. External Input

The pyramidal population is the only one subject to an external input ξ (see Appendix A.2 for full model equations). Hence, it will be termed *perturbed cortical population* from here on and its cortical column will be termed *perturbed cortical column*. The external input is considered to be a transient 100 ms increase in the mean of the white noise ϕ (see Table 2 for parameter values). It is modeled as a square function (see Figure 1) of $1\;{\mathrm{ms}}^{-1}$ amplitude.

### 2.6. Model Extension to Two Cortical Columns

We have extend the model to two bidirectionally connected cortical columns (see Figure 1B). Besides the perturbed cortical column, now we model a second cortical column that does not directly receive the external input ξ and will be from here on termed *unperturbed cortical column* and its pyramidal population will be termed *unperturbed cortical population*. The rest of parameters are the same for the perturbed and unperturbed cortical columns. We consider that the connectivity between the two cortical columns is symmetric and excitatory due to the longer axons of pyramidal neurons as compared to the short-range axons of inhibitory neurons [30,36,37,38,39,40,41]. The average AMPAergic conductance of excitatory synapses between the two cortical columns will be referred here as inter-conductance, as opposed to the intra-conductance of each single cortical column. The average GABAergic conductance on pyramidal and inhibitory populations are here increased to counterbalance this inter-cortical column coupling that introduces more excitation to the pyramidal and inhibitory populations. By doing so, we keep the steady state value of $V_{p}$ and $V_{i}$ in the two-cortical-column scenario equal to the values in the one-cortical-column scenario both during the Up states of NREM sleep and wakefulness (see Table 2 for parameter values and Appendix B.2 for the dynamical constraints on the increased average GABAergic conductance during NREM sleep and wakefulness in the two-cortical-column model). Therefore, the second-order differential equations for AMPAergic postsynaptic responses in each cortical column due to the inter-cortical column coupling are as follows:

(11) $$\begin{array}{cc} {\ddot{s}}_{pp^{\prime }}^{\;\mathrm{inter}} & =\gamma_{p^{\prime }}^{2}\;\left(N_{pp^{\prime }}\;Q_{p^{\prime }}\left(V_{p^{\prime }}\right)-s_{pp^{\prime }}^{\;\mathrm{inter}}\right)\;-2\gamma_{p^{\prime }}\;{\dot{s}}_{pp^{\prime }}^{\;\mathrm{inter}}, \end{array}$$

(12) $$\begin{array}{cc} {\ddot{s}}_{ip^{\prime }}^{\;\mathrm{inter}} & =\gamma_{p^{\prime }}^{2}\;\left(N_{ip^{\prime }}\;Q_{p^{\prime }}\left(V_{p^{\prime }}\right)-s_{ip^{\prime }}^{\;\mathrm{inter}}\right)\;-2\gamma_{p^{\prime }}\;{\dot{s}}_{ip^{\prime }}^{\;\mathrm{inter}}, \end{array}$$

where $N_{kp^{\prime }}$ is the mean number of synaptic connections from a presynaptic pyramidal population $p^{\prime }$ in one cortical column to postsynaptic populations k=p,i in the other cortical column (see Appendix A.3 for full model equations).

We introduce the parameter β as the ratio of the average AMPAergic inter-conductance to the average AMPAergic intra-conductance. Therefore, the AMPAergic current to the postsynaptic population *k* in each cortical column is as follows:

(13) $$I_{\mathrm{AMPA}}^{k}={\bar{g}}_{\mathrm{AMPA}}^{\;k}\;s_{kp^{\prime }}\;\left(V_{k}-E_{\mathrm{AMPA}}\right)\;+\;\beta \cdot {\bar{g}}_{\mathrm{AMPA}}^{\;k}\;s_{kp^{\prime }}^{\;\mathrm{inter}}\;\left(V_{k}-E_{\mathrm{AMPA}}\right),$$

where *k* is either the pyramidal or inhibitory postsynaptic population of either cortical column.

We quantify information propagation to the unperturbed population as a function of β and ${\bar{g}}_{\mathrm{AMPA}}^{\;k}$. First, we increase β from 2 to 5 in steps of one unit while ${\bar{g}}_{\mathrm{AMPA}}^{\;k}=2$ is kept constant. To counterbalance the increase in excitation to pyramidal and inhibitory populations due to these changes in β, we increase the average GABAergic conductance on pyramidal and inhibitory populations (see Table 4 for parameter values).

Next, we repeat the same approach for two other values of ${\bar{g}}_{\mathrm{AMPA}}^{\;k}=$ 6 and 10. In each case, β is varied within the aforementioned values. Again, to counterbalance the increase in excitation to the pyramidal and inhibitory populations, we increase the average GABAergic conductance on pyramidal and inhibitory populations of both cortical columns (seeTable 5 and Table 6 for ${\bar{g}}_{\mathrm{GABA}}^{\;k}$ values at ${\bar{g}}_{\mathrm{AMPA}}^{\;k}=$ 6 and 10, respectively).

### 2.7. Numerical Integration

The model was implemented and run in Python, using a stochastic Heun method [47] with a step size of 0.1 ms. The code is available at github [48]. Each simulation was run independently, choosing the initial conditions of all variables at random from a uniform distribution. Averages are obtained across 500 independent trials of length 10 s after discarding the initial 10 s from the simulation onset to eliminate transient dynamics towards the stable solution.

### 2.8. Data Analysis

All data analyses were carried out offline with Python. We simulated six signals from each cortical column: LFP, firing rate and average membrane potential signals from both pyramidal and inhibitory populations.

#### 2.8.1. LFP Definition

The LFP signal is defined as the sum of the absolute value of AMPAergic and GABAergic synaptic currents to cortical population *k* of either cortical column [49,50]. The action potentials are fast events of less than 10 ms that are attenuated by the cortical tissue and, therefore, do not contribute to the LFP signals.

(14) $$\mathrm{LFP}=|I_{\mathrm{AMPA}}^{k}\left|+\right|I_{\mathrm{GABA}}^{k}|.$$

#### 2.8.2. Analysis of Amplitude-Frequency Content of LFP Signal

In each trial, we removed the temporal mean of the LFP of prestimulus intervals of either cortical column (from the beginning of simulation up to five seconds later). We used the prestimulus intervals of either cortical column to analyze the amplitude-frequency content during NREM and wakefulness states and the poststimulus intervals (from external input onset up to five seconds later) to analyze the evoked responses in either cortical column due to the external input.

To asses the spread of the spontaneous LFP values, we computed the distribution of the LFP during the prestimulus interval of both cortical columns for each brain state separately with a bin size of 0.5 mV. To locate the peaks of the bimodal LFP distribution during NREM sleep, the distribution for pyramidal (inhibitory) population was first smoothed with a Gaussian kernel, $\exp(-\frac{x^{2}}{2c^{2}})$, where *c* is equal to $5\sqrt{5}$ mV (5 mV). The half distance between the Up and Down peaks in the LFP distribution during NREM sleep was used as a threshold to classify these events in NREM sleep. Events with the LFP signal higher than the threshold were classified as Up states, otherwise they were labeled as Down states. Then, the distribution of the firing rate, in 0.5 Hz bins, and the average membrane potential, in 0.5 mV bins, were computed separately for the Up and Down states. Distributions were normalized by the total number of events during NREM sleep. Besides, the distribution of the firing rate and the average membrane potential during wakefulness were computed and normalized in the same way.

In order to compute the frequency content of the LFP signal during NREM sleep and wakefulness, we computed its spectrogram. To do so, we first windowed the LFP signals in 2 s length intervals with a 90% overlap within which the LFP is considered quasi-stationary [51]. Then, each window was tapered with a Hann function to control for the spectral leakage, and we computed the discrete Short-Time Fourier Transform (STFT). Next, the power spectral density (PSD) on each time window was obtained from the amplitude of the STFT. Because NREM sleep and wakefulness were independently simulated and were not time-locked events, we averaged the PSD over all time windows [52]. To better observe the wide dynamic range of the LFP, we plotted the spectrogram in units of logarithmic decibels (dB), defined as:

(15) $$\mathrm{dB}=\log_{10}\left(\frac{P}{P_{r}}\right),$$

where *P* is the averaged PSD and $P_{r}$ the reference PSD, set at 1 ${\left(\mathrm{mV}\right)}^{2}/\mathrm{Hz}$. Finally, the spectrogram of the LFP signals were averaged over 500 simulations for each brain state.

The distribution of the log ratio between high (>30 Hz) and low (<4 Hz) frequency bands of the prestimulus intervals was computed to further verify the different dynamics between NREM and wakefulness.

#### 2.8.3. Statistical Test for Significance of the Evoked Response

We tested the significance of the evoked responses in the poststimulus intervals in the one-cortical-column and the two-cortical-column scenario. First, we tested whether the postistimulus signals differed significantly from the spontaneous activity captured in the prestimulus intervals during NREM sleep and wakefulness, separately. This test allowed us to quantify the emergence of a response to the external input. Second, we tested whether the postistimulus signals differed significantly between brain states. This test allowed us to quantify the sensitivity of the response to the external input to the spontaneous dynamics.

In the case of the two cortical columns scenario, we verified whether the postistimulus signals differed significantly from the spontaneous activity in the perturbed population to maintain the same conditions of the single cortical column. For the unperturbed population, the same test allowed us to quantify the propagation of the response.

These significance tests were run independently for all signals in the one- and two-cortical-column scenario. The statistical procedure was carried out using a temporal clustering nonparametric test [53] to control for family-wise error in multiple comparisons of temporal *t*-tests. Since the number of simulations was large (500 simulations) for the Central Limit Theorem to hold, we computed an independent *t*-test value (*t*-value) at every time point as $t=\left({\bar{x}}_{1}-{\bar{x}}_{2}\right)/\left(s\sqrt{2/n}\right)$, where ${\bar{x}}_{1}$ and ${\bar{x}}_{2}$ are the averages across simulations of the signals that are being compared against the null hypothesis of no statistical difference between them. *s* is the sample standard deviation, whose variance is $s^{2}=\left(\left(n-1\right)s_{1}^{2}+\left(n-1\right)s_{2}^{2}\right)/\left(2n-1\right)$. $s_{1/2}^{2}$ is the variance of variable $x_{1/2}$ across simulations. Statistical significance is set at *p*-values below 0.01, which corresponds to a two-tailed critical *t*-value = ±2.58, for n=500. We computed the area of all clusters made of consecutive time points (referred as t-cluster statistic) where $|{\bar{x}}_{1}-{\bar{x}}_{2}|\geq$2.58 and sorted them in descending order. Clusters with an area below the largest t-cluster statistic computed from the prestimulus activity were disregarded. Note that when $x_{1/2}$ represent the prestimulus and poststimulus interval from the same population and brain state, this largest t-cluster is obtained by splitting in half the prestimulus activity $x_{2}$.

Then, we built a null distribution for each of the remaining ordered t-cluster statistics as follows. We shuffled the simulations of ${\bar{x}}_{2}$ with respect to signal ${\bar{x}}_{1}$ and extracted again the t-cluster statistics, sorting them in descending order. We shuffled the simulations until we reached 1000 permuted clusters for the smallest t-cluster statistic. Each null distribution was assigned to the observed t-cluster statistic with the same ordinal position. Finally, for each observed t-cluster statistic, we computed its significance from the proportion of clusters that were larger than the observed one in the corresponding null distribution.

#### 2.8.4. Quantification of Information Propagation

We quantified the propagation of the external input from the perturbed to the unperturbed population as a function of β and ${\bar{g}}_{\mathrm{AMPA}}^{\;k}$. When ${\bar{x}}_{1,2}$ corresponds to the firing rate signals of the unperturbed population during the poststimulus/prestimulus interval, the magnitude and length of the first t-cluster statistic after stimulus onset were used to quantify the amount of information propagation. A bigger and longer t-cluster statistic was indicative of a higher transmission of information to the unperturbed population. The magnitude (in s) and length (in ms) of the first t-cluster statistic were plotted as a function of β and ${\bar{g}}_{\mathrm{AMPA}}^{\;k}$.

## 3. Results

In the following, we first present the results for the one-cortical-column model. We show that the model reproduces the dynamical features of NREM sleep and wakefulness, given the parameters shown in Table 2. Then, we show that in response to a transient increase in the input firing rate, the model reproduces the experimental results obtained by Nir and colleagues [7]. In particular, the amplitude of the response is larger and followed by a deeper negative deflection, corresponding to a poststimulus suppression of neuronal activity, in NREM sleep as compared to wakefulness.

Second, we extend the results to two coupled cortical columns and we show that the dynamics of each brain state are preserved in this scenario. Then, we study the propagation of the external input from the perturbed cortical column to the unperturbed cortical column. Finally, we show that the upscaling of the average AMPAergic conductance during wakefulness is not sufficient for propagating the response to the external input. Instead, a higher increase in the AMPAergic conductance of the inter-cortical excitatory connectivity with respect to the intra-cortical connectivity is necessary to transmit information.

All the figures shown in the main text refer to the pyramidal population. The corresponding figures for the inhibitory population are provided in the supplementary material (Figures S1, S2, S4–S7 and S9).

### 3.1. One-Cortical-Column Model

#### 3.1.1. Spontaneous Activity across Brain States

**Figure 2:**
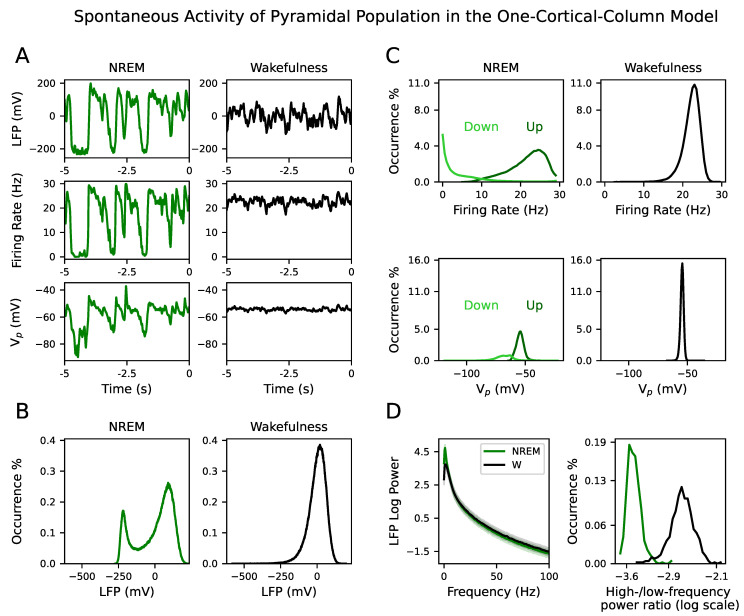
Spontaneous activity (ξ=0) during NREM sleep and wakefulness in the one-cortical-column model. (**A**) Simulated signals from one random simulation showing large amplitude fluctuations during NREM sleep between Up and Down states (left column). Lower amplitude fluctuations appear during wakefulness (right column). The first, second and third row correspond to LFP, firing rate and $V_{p}$ signal, respectively. (**B**) The distribution of the LFP signals is bimodal during NREM sleep and unimodal during wakefulness. The half distance between the two peaks of the NREM distribution is used to extract Up and Down events from the LFP. (**C**) Top row: The distribution of the firing rate during Up states (dark green) shows the persistent activity mode, while the Down state distribution (light green) is a silent activity mode. The distribution of firing rate during wakefulness shows a unimodal distribution. The distribution during wakefulness and Up states have similar means. Bottom row: The distribution of the $V_{p}$ signal during Up (dark green) and Down (light green) events differ. It has a more depolarized mean value during Up states (close to the mean value during wakefulness) than Down states. The bigger area of the distributions of Up events indicates that they are more prevalent than Down events in the modeled NREM sleep. (**D**) Spectral content of LFP signals during NREM sleep and wakefulness. Left: Average power spectrum density of LFP signals during NREM sleep (green) and wakefulness (black). The shaded area corresponds to the standard deviation of the mean of PSD over 500 simulations during NREM sleep and wakefulness. Right: Distribution of high-/low-frequency (where high is above 30 Hz and low is below 4 Hz) power ratio in the LFP during NREM sleep (green) and wakefulness (black).

Spontaneous activity differs between NREM sleep and wakefulness. In the model, it was obtained by potentiating ${\bar{g}}_{\mathrm{AMPA}}$ (see Section 2.4). All signal types—LFP, firing rate and membrane potential—showed larger fluctuations during NREM sleep than during wakefulness (see one example of a simulated signal for LFP, firing rate and $V_{p}$ in NREM sleep and wakefulness in Figure 2A). The distribution of the LFP signals during NREM sleep is bimodal, whereas for wakefulness is unimodal (see Figure 2B) in agreement with the occurrence of Up and Down states in the former case [3,4,5,6,7,8].

The firing rate and $V_{p}$ signals during NREM sleep were split into Up and Down states (see Section 2.8.2). The distribution of the firing rate during Down states peaks at zero, confirming that it is a quasi-silent activity mode of the NREM dynamics. On the contrary, the firing rate during Up states peaks at a higher value, corresponding to persistent activity (see the top row in Figure 2C). This bimodality was also present in the $V_{p}$ signal, where its mean value during Up states had a more depolarized value than the Down state (see the bottom row in Figure 2C). Note that the distribution of firing rates and $V_{p}$ during wakefulness is closer to the Up state distribution than to the Down state distribution.

LFP signals had more spectral power within the delta (0.5–2 Hz) and SWA frequency band (below 4 Hz) during NREM sleep than during wakefulness; whereas gamma power (>30 Hz) was highest in wakefulness (see left panel in Figure 2D). The distribution of high-/low-frequency (where high is above 30 Hz and low is below 4 Hz) power ratio in the LFP confirmed the spectral separation between NREM sleep and wakefulness (see right panel in Figure 2D). These results were confirmed in the inhibitory population as well (see Figure S1). Therefore, the simulated spontaneous activity in the NREM sleep and wakefulness parameter space exhibited the experimental dynamical features characteristic of each brain state.

#### 3.1.2. Evoked Responses in the One-Cortical-Column Model

**Figure 3:**
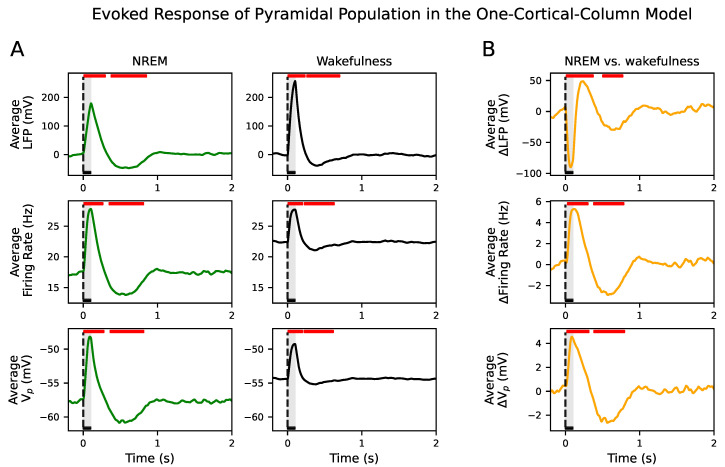
Evoked response in the one-cortical-column model. (**A**) Average LFP, firing rate and $V_{p}$ across 500 simulations for NREM sleep (left column) and wakefulness (right column). Red horizontal bars show the location and length of significant t-cluster statistics when comparing the poststimulus versus the prestimulus intervals. In all signals, responses to the external stimulus consist of one positive and one negative significant deflection. The positive (p<0.0006) and negative (p<0.001) deflections are significant in NREM and wakefulness. (**B**) Average difference ΔLFP, Δfiring rate and $\Delta V_{p}$ between NREM sleep and wakefulness across 500 simulations. Red horizontal bars show the location and length of significant t-cluster statistics when comparing the poststimulus intervals during NREM sleep with wakefulness. The positive (p<0.0005) and negative (p<0.001) deflections in all signals are significant. The amplitude of both deflections are higher in NREM sleep than in wakefulness in the firing rate and $V_{p}$. The opposite happens in the LFP signal. In all cases, the dashed vertical line, aligned at zero, indicates stimulus ξ onset. Stimulus duration is represented by a black horizontal line and light gray area.

An external input consisting of an increase of $1\;{\mathrm{ms}}^{-1}$ in the mean of the noise term ϕ during 100 ms is applied to the pyramidal population. The evoked responses in poststimulus intervals were significantly different from the spontaneous activity in prestimulus intervals in all signal types during NREM sleep and wakefulness (see Figure 3A). Input responses showed significant deviations from spontaneous activity in two time intervals. The first period corresponded to a positive deflection of the signals above prestimulus values (corresponding to a poststimulus neuronal activity in response to the external input), while the second interval corresponded to a negative deflection well beyond stimulus offset (corresponding to a poststimulus suppression of neuronal activity) (see Figure 3A).

We also compared the evoked responses between brain states (see Figure 3B). Since the spontaneous value in wakefulness is higher than in NREM sleep, we removed its temporal average to compare the amplitude of the positive and negative deflections of the firing rate and $V_{p}$ signals. We observed that those deflections were significantly larger in NREM sleep than during wakefulness. Therefore, both the response to the external input and later suppression of neuronal activity were higher in NREM sleep than in wakefulness. However, the positive and negative deflections in the evoked response captured in the LFP were higher in wakefulness than in NREM sleep. These results regarding the firing rate and the negative deflections of the LFP are in agreement with the reported experimental behavior in [7]. These results were confirmed in the inhibitory population as well (see Figure S2).

### 3.2. Two-Cortical-Column Model

The dynamics of each brain state were preserved after coupling two cortical columns (see Figure S3) although the excitatory inter-connectivity caused a decrease in the occurrence of Down states and a depolarization of $V_{p}$ during the Up states of NREM sleep compared to the case of a single cortical model (see Figure S3B,C).

LFP signals in NREM sleep carried more spectral power within the delta and SWA frequency band. Gamma power was highest in wakefulness. Further analysis of high-/low-frequency power ratio in the LFP signals confirmed the spectral separation between NREM sleep and wakefulness in the two cortical model scenario (see Figure S3D). Thus, the coupling between the two cortical columns did not change qualitatively the intrinsic dynamics of each brain state. Results were consistent in the inhibitory population as well (see Figure S4).

#### 3.2.1. Evoked Responses in the Two-Cortical-Column Model

**Figure 4:**
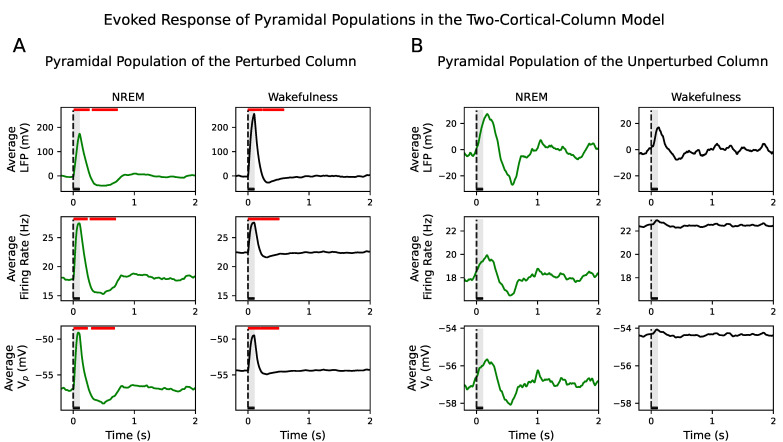
Evoked responses in the two-cortical-column model where β=1 and ${\bar{g}}_{\mathrm{AMPA}}=2$. (**A**) Average LFP, firing rate and $V_{p}$ across 500 simulations for NREM sleep (left column) and wakefulness (right column) in the perturbed pyramidal population. Red horizontal bars show the location and length of significant t-cluster statistics when comparing the poststimulus versus the prestimulus intervals. In all signals, as in Figure 3A, responses to the external stimulus consist of one positive and one negative significant deflection. The positive (p<0.0007) and negative (p<0.001) deflections are significant in NREM and wakefulness. Note that the coupling between the two cortical columns does not change the significance of positive and negative deflections in the evoked responses. (**B**) Results are as in panel (**A**) but for the case of the unperturbed pyramidal population. The evoked responses do not show significant deflections during NREM sleep and wakefulness in the LFP signal, neither in the firing rate nor in $V_{p}$, where the increase in activity is not significantly higher than the spontaneous activity. In all cases, the dashed vertical line, aligned at zero, indicates stimulus ξ onset. Stimulus duration is represented by a black horizontal line and light gray area.

The evoked responses of the perturbed population in the perturbed cortical column in the two-cortical-column scenario were maintained, i.e., they were significantly different from the spontaneous activity during the prestimulus interval in all signal types during NREM sleep and wakefulness (see Figure 4A). Similarly to Figure 3A, two significant temporal clusters appeared following stimulus onset.

On the contrary, the evoked responses in the unperturbed population captured in the firing rate and $V_{p}$ signals were not significantly different from the spontaneous activity neither during NREM sleep nor during wakefulness (Figure 4B). In the latter case, that was so despite the increase in the average AMPAergic conductance, ${\bar{g}}_{\mathrm{AMPA}}$, affecting the excitatory inter- and intra-connections. This upscaling of the conductance of excitatory synapses that allowed us to move the system from NREM to wakefulness dynamics, was not able to produce a significant LFP response, neither in the firing rate nor in the membrane potential, of the unperturbed population in response to the perturbed population (see right column in Figure 4B). These results were confirmed in the inhibitory populations as well (see Figure S5).

#### 3.2.2. Information Propagation as a Function of β and ${\bar{g}}_{\mathrm{AMPA}}$

**Figure 5:**
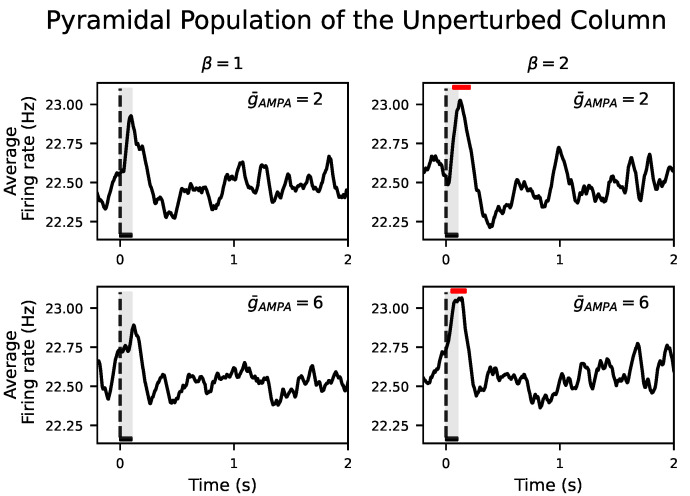
Evoked responses in the two-cortical-column model for β=1,2 and ${\bar{g}}_{\mathrm{AMPA}}=2,\;6$. Average across 500 simulations of the firing rate of the unperturbed pyramidal population. Red horizontal bars show the location and length of significant t-cluster statistics when comparing the poststimulus versus the prestimulus interval. Firing rate of the unperturbed population shows a positive deflection after stimulus onset in all cases. Increasing ${\bar{g}}_{\mathrm{AMPA}}$ from 2 to 6 evokes a significant response (p<0.003) in the unperturbed population only if β is also increased from 1 to 2. In all cases, the dashed vertical line, aligned at zero, indicates stimulus ξ onset. Stimulus duration is represented by a black horizontal line and light gray area.

**Figure 6:**
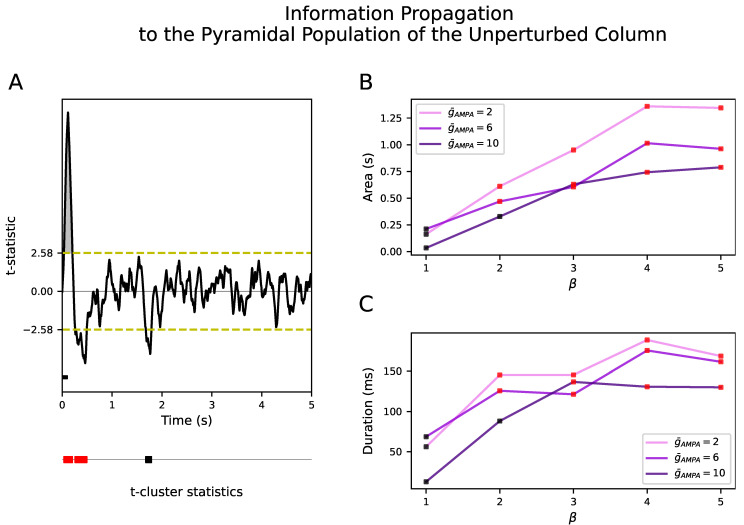
Information propagation as a function of β and ${\bar{g}}_{\mathrm{AMPA}}$. (**A**) Example of extraction of t-cluster statistics when comparing the evoked responses in firing rate signals with the spontaneous activity in the prestimulus intervals at ${\bar{g}}_{\mathrm{AMPA}}=2$ and β=5. The yellow horizontal dashed line defines the critical t-value. The concatenation of consecutive t-values above and below this threshold that forms the t-cluster statistics is shown as rectangles in the bottom line. Significant and non significant t-cluster statistics are indicated in red and black, respectively. The area and duration of the first t-cluster statistic, shown in gray, are used to quantify response propagation to the unperturbed population. Stimulus duration is represented by a black horizontal line. (**B**) Changes in the area of the first significant t-cluster statistic in seconds (y-axis) with respect to β (x-axis). (**C**) Changes in the length of the first significant t-cluster statistic in milliseconds (y-axis) with respect to β (x-axis). Violet, dark violet and indigo correspond to ${\bar{g}}_{\mathrm{AMPA}}=2$, ${\bar{g}}_{\mathrm{AMPA}}=6$ and ${\bar{g}}_{\mathrm{AMPA}}=10$, respectively. Figures show that the propagated information to the unperturbed population depends on β. Increasing ${\bar{g}}_{\mathrm{AMPA}}$ from 2 to 10 and keeping β=1 does not lead to a significant cluster in the unperturbed population (black squares). Increasing β from 1 to 3 shows a significant cluster (red squares) in all values of ${\bar{g}}_{\mathrm{AMPA}}$. In the case of ${\bar{g}}_{\mathrm{AMPA}}=2$, the t-cluster statistics are significant (p<0.001) for β= 2, 3, 4 and 5. In the case of ${\bar{g}}_{\mathrm{AMPA}}=6$, the t-cluster statistics are significant (p<0.003) for β>1. In the case of ${\bar{g}}_{\mathrm{AMPA}}=10$, the t-cluster statistics are significant (p<0.001) for β>2.

Figure 4B showed that the response of the perturbed population to the external input was not propagated to the unperturbed population in neither NREM sleep nor wakefulness. During NREM sleep, we aim at reproducing such local responses in agreement with the experiments [7,9]. However, this is at odds with a wider spread of electrical activity during wakefulness. Even increasing ${\bar{g}}_{\mathrm{AMPA}}$ from 2 to 6 for all inter- and intra-excitatory synapses (corresponding to an inter-/intra-conductance ratio of cortical excitatory synapses β=1) was not able to propagate the input to the unperturbed column. Interestingly, a selective increase of only the inter-excitatory synapses (β=2) showed a significant positive deflection at the firing rate signal in the unperturbed cortical column. This corresponds to an increase of neural activity after the onset of the external input with respect to the spontaneous activity for both ${\bar{g}}_{\mathrm{AMPA}}=2$ and ${\bar{g}}_{\mathrm{AMPA}}=6$ (see Figure 5). These results were confirmed in the inhibitory populations as well (see Figure S6). The size and duration of such a positive deflection improved as a function of β in all signal types (see Figure 6B,C for the firing rate signal).

Our results show that keeping β=1 does not trigger a significant response in the unperturbed population even when potentiating ${\bar{g}}_{\mathrm{AMPA}}$ to 10, at which value no significant t-cluster statistic still appears (see black squares at β=1 in Figure 6B,C). This non-selective increase of ${\bar{g}}_{\mathrm{AMPA}}$ only gives rise to a larger amplitude fluctuation in the LFP but not in the firing rate nor in $V_{p}$ (results not shown here). Importantly, a selective increase of ${\bar{g}}_{\mathrm{AMPA}}$ for the inter-excitatory connections over the intra-excitatory connections, quantified by β, is required to enlarge the size and duration of the positive deflection. Results were consistent in the inhibitory population as well (see Figure S7). This dependence of information propagation on β still held when performing a Bonferroni corrected *t*-test rather than a temporal clustering nonparametric test (see Figures S8 and S9 for pyramidal and inhibitory population, respectively).

The observed dependence of information propagation on β is due to the fact that the input–output function of any system depends on the signal to noise ratio (SNR). In the case of neural populations, the local synaptic currents would play the role of a stochastic signal and the currents activated by the external input would form the signal. Therefore, the non-selective increase of synaptic excitatory afferents within and between cortical columns from NREM sleep to wakefulness would not change the SNR. In order to boost signal transmission, synaptic potentiation needs to be larger for the synapses between cortical columns than for the synapses within cortical columns.

## 4. Discussion

There is compelling experimental evidence that auditory stimuli elicit firing responses in the primary auditory cortex that are similar in NREM sleep and wakefulness [7,9]. This poses a challenge to the understanding of auditory perception across the wake–sleep cycle because sounds seem to bypass the thalamus in both brain states. However, the observation that neuronal responses to either auditory stimuli [7,8,9,11,12] and non-sensory stimuli [15,16] are elicited in areas located at distances from the stimulation site that are larger in wakefulness than in NREM sleep opens the door to a new hypothesis. In particular, researchers have suggested that it is the propagation of sounds throughout the cortex that is compromised during sleep, given that a neuronal response is detected in primary brain areas at the initial stages of auditory processing. This hypothesis is supported by the fact that effective connectivity during sleep is reduced with respect to wakefulness, a feature that has also been explored in large-scale models [13,39].

Here, we have used a computational approach to understand how input propagation is disrupted, beyond primary sensory encoding, by tuning the conductance of synaptic connections. By means of a neuronal mass model operating in two distinct brain dynamics, NREM sleep and wakefulness, we investigate the response of a neural system composed of two cortical columns to an external perturbation. Our simulated signals—LFP, firing rate and membrane potential—have a bimodal distribution in the parametric space belonging to NREM dynamics, which is indicative of the alternation between Up and Down states and the presence of slow oscillations. The higher power at low frequencies in NREM versus the higher power at higher frequencies in wakefulness of the LFP also validates the existence of these two different dynamical regimes.

Few models exhibiting both regimes have been published up to date [26,27,28,29,54]. Despite the fact that any computational model with multiple parameters exhibits a rich variety of bifurcations, we show that the sleep–waking transition can be obtained with the minimum set of possible changes. Naturally, the simplest solution is to vary a single parameter, in contrast with the proposal of [27,31]. Here, an increase in the AMPAergic conductance ${\bar{g}}_{\mathrm{AMPA}}^{\;k}$ suffices to move the system from NREM-like to waking-like dynamics. This is so because in the model, the membrane potential relaxes back to its steady state value producing damped oscillations in response to a perturbation for the parameter space of the NREM state (see Figure S10) due to the presence of a stable focus [55]. This behavior generates the slow oscillation in the presence of noise. Moreover, this change is consistent with the waxing and waning of synaptic strengths across the wake–sleep transition that SHY proposes [17,18,19,20,21,22,23,24,25].

In this study, the connectivity between the two cortical columns is excitatory and symmetric, but only one of the pyramidal populations receives a transient external input. We investigate how this perturbation is transmitted to the other column in such a way that, in agreement with the aforementioned experiments, its response is enhanced in the awake state when compared to NREM. Previous computational work [30] also aimed at reproducing the distinct propagation patterns that arise across the SWC, obtaining similar results to the ones described here. In particular, a wider propagation of the external perturbation occurs during wakefulness than during NREM sleep. In our case, this is quantified by the response of the unperturbed population, whereas [30] uses the perturbational complexity index (PCI) [56]. However, the set of parameters’ changes used by [30] focus on an adaptation current that weakens during wakefulness, which in our opinion is less experimentally justified. Moreover, the authors showed that during wakefulness the ratio between poststimulus and prestimulus firing rate can be greater in an area that is not directly stimulated than in the stimulation site, which requires further experimental verification.

We show that when the conductance of the excitatory synapses, ${\bar{g}}_{\mathrm{AMPA}}^{\;k}$, within a cortical column and between neighboring cortical columns are equal, there is no propagation of the input to the unperturbed cortical column. Propagation only occurs when the synaptic conductance between cortical columns is selectively boosted with respect to the synaptic conductance within cortical columns, a ratio that we have quantified with the parameter β. It is important to note that this difference between the inter- and intra-AMPAergic conductance does not directly result in a higher AMPAergic external than local synaptic afferent current. In fact, in the model, local synaptic currents surpass external ones both during NREM sleep and wakefulness. However, during wakefulness, this synaptic imbalance increases. Therefore, our results suggest that the synaptic homeostasis hypothesis should be heterogeneously applied, that is, that synaptic potentiation has to be spatially organized and distant synaptic conductances have to be scaled with respect to the local ones. Although this result needs experimental validation, it has been reported that, in the cortex, perforated synapses enlarge their axon–spine interface after waking relative to sleep, in contrast to the lack of change found in non-perforated synapses [25]. This morphological change specific to perforated synapses also speaks for their major role in synaptic plasticity and the selective implementation of synaptic homeostasis that we propose.

Despite the fact that our model cannot account for whole brain interactions and is restricted to the dialogue between two networks, our results are derived from physiological assumptions and reproduce verified experimental observations. A more complex connectivity diagram comprising other cortical and subcortical structures could, nonetheless, provide spatial information about stimulus propagation. Subcortical structures, such as the brainstem and basal forebrain, are the substrate for the changes in the concentration of neuromodulators that occur throughout the sleep–weak cycle [57]. These neuromodulators target vesicular release at the presynaptic site or transmitter receptors at the postsynaptic site and alter synaptic strength [58,59]. We have phenomenologically implemented their role by tuning synaptic conductances but the precise effect of these substances (serotonin, acetylcholine, histamine, etc.) on target brain areas is out of the scope of this study, partly because the exact microcircuitry of the ascending arousal system is still unknown.

It is important to note that we have excluded REM sleep from our work, which is a short but relevant stage of sleep that follows NREM epochs. The LFP and EEG dynamics recorded during REM sleep show similar features to wakefulness. Since firing responses in the primary auditory cortex are preserved across the SWC, including REM sleep [7,8], computationally, we could use the same set of parameters to model REM than wakefulness at a single cortical column level. However, tracking these responses up to the perirhinal cortex, shows that their amplitude is attenuated from wakefulness to NREM sleep, and has intermediate values in REM sleep [8]. In our model, this could be replicated by using a β value for REM sleep smaller than for wakefulness (see Figure 3B,C). However, experimentally, SHY has only proven to be true when comparing wakefulness and NREM sleep [25]. Therefore, we need to be cautious before extending our conclusions to REM sleep.

Despite the limitations of our model, our work opens the door to explore which rules need to be implemented to reproduce experimental data and guide computational principles. This is particularly important while we still lack a clear understanding of how inputs scattered along the wide dendritic tree of a pyramidal neuron are added to combine synaptic sources at short and long distances with respect to the soma.

## 5. Conclusions

Sleep and wakefulness are distinguished by distinct behavioural, electrophysiological and molecular features. It is still unclear how these biological layers are causally connected. Here, we have explored one particular relationship between two experimentally observed changes that occur across the SWC: the potentiation of excitatory synapses and the broad distribution of incoming information throughout the cortex that takes place during wakefulness. The former happens during learning and strengthens synaptic connections, a process that is compensated during NREM sleep to enable synaptic homeostasis. The later evidence may speak for a cortical gating mechanism by which signals coming from the external world only reach higher processing brain areas during wakefulness and could possibly explain the emergence of consciousness. Our computational model links both phenomena by showing that the potentiation of excitatory conductances not only triggers a dynamic change of the electrical activity of the neuronal networks, but also of the propagation pattern across them. However, we predict that, in order for a spatially wider response to occur during wakefulness, it is necessary that such potentiation occurs preferentially between distinct networks over local and recurrent connections.

## Figures and Tables

**Table 1:** Parameter description for the neural mass model.

Symbol	Description
$Q_{k}^{\max}$	Maximal firing rate of population *k*
$\theta_{k}$	Firing rate threshold of population *k* (sigmoid function half activation)
$\sigma_{k}$	Inverse neural gain of the sigmoid function of population *k*
$\tau_{k}$	Membrane time constant of population *k*
$C_{m}$	Membrane capacitance in the HH model
ϕ	Stochastic Gaussian process
$N_{kk^{\prime }}$	Mean number of synaptic connections from presynaptic
	population $k^{\prime }$ to postsynaptic population *k*
$\gamma_{m}$	Time constant of the postsynaptic response of synapse type *m*
${\bar{g}}_{X}^{\;k}$	Average X-ergic conductance on population *k*
$E_{X}$	Reversal potential of the X-ergic current
${\bar{g}}_{\mathrm{KNa}}$	Average conductance of sodium-dependent potassium channel
$E_{K}$	Nernst reversal potential of potassium channel
$\tau_{\mathrm{Na}}$	Time constant of sodium extrusion
$\alpha_{\mathrm{Na}}$	sodium influx through population firing rate
$R_{\mathrm{pump}}$	Strength of the sodium pump
${\mathrm{Na}}_{\mathrm{eq}}$	Resting state sodium concentration equilibrium

**Table 2:** Parameter values for the one-cortical-column model.

Symbol	NREM	W	Unit
$Q_{p}^{\max}$	0.03	0.03	${\mathrm{ms}}^{-1}$
$Q_{i}^{\max}$	0.06	0.06	${\mathrm{ms}}^{-1}$
$\theta_{p}$, $\theta_{i}$	−58.5	−58.5	mV
$\sigma_{p}$	6.7	6.7	mV
$\sigma_{i}$	6	6	mV
$\tau_{p}$, $\tau_{i}$	30	30	ms
$C_{m}$	1	1	$\mu F/{\mathrm{cm}}^{2}$
ϕ	1.8	1	${\mathrm{ms}}^{-1}$
$N_{pp}$	160	160	-
$N_{ip}$	40	40	-
$N_{pi}$	160	160	-
$N_{ii}$	40	40	-
$\gamma_{p}$	$70\times 10^{-3}$	$70\times 10^{-3}$	${\mathrm{ms}}^{-1}$
$\gamma_{i}$	$58.6\times 10^{-3}$	$58.6\times 10^{-3}$	${\mathrm{ms}}^{-1}$
${\bar{g}}_{\mathrm{AMPA}}^{\;p}$	1	2	ms
${\bar{g}}_{\mathrm{AMPA}}^{\;i}$	1	2	ms
${\bar{g}}_{\mathrm{GABA}}^{\;p}$	1	2.294	ms
${\bar{g}}_{\mathrm{GABA}}^{\;i}$	1	2.313	ms
$E_{\mathrm{AMPA}}$	0	0	mV
$E_{\mathrm{GABA}}$	−70	−70	mV
$E_{L}^{p}$	−66	−66	mV
$E_{L}^{i}$	−64	−64	mV
${\bar{g}}_{\mathrm{KNa}}$	1.9	1.9	$\mathrm{mS}/{\mathrm{cm}}^{2}$
$E_{K}$	−100	−100	mV
$\tau_{\mathrm{Na}}$	1.7	1.7	ms
$\alpha_{\mathrm{Na}}$	2	2	mM·ms
$R_{\mathrm{pump}}$	0.09	0.09	mM
${\mathrm{Na}}_{\mathrm{eq}}$	9.5	9.5	mM

**Table 3:** Parameter values for the two-cortical-column model.

Symbol	NREM	W	Unit
$Q_{p}^{\max}$, $Q_{p^{\prime }}^{\max}$	0.03	0.03	${\mathrm{ms}}^{-1}$
$Q_{i}^{\max}$, $Q_{i^{\prime }}^{\max}$	0.06	0.06	${\mathrm{ms}}^{-1}$
$\theta_{p}$, $\theta_{i}$, $\theta_{p^{\prime }}$, $\theta_{i^{\prime }}$	−58.5	−58.5	mV
$\sigma_{p}$, $\sigma_{p^{\prime }}$	6.7	6.7	mV
$\sigma_{i}$, $\sigma_{i^{\prime }}$	6	6	mV
$\tau_{p}$, $\tau_{i}$, $\tau_{p^{\prime }}$, $\tau_{i^{\prime }}$	30	30	ms
$C_{m}$	1	1	$\mu F/{\mathrm{cm}}^{2}$
ϕ	1.8	1	${\mathrm{ms}}^{-1}$
$N_{pp}$, $N_{p^{\prime }p^{\prime }}$	160	160	-
$N_{ip}$, $N_{i^{\prime }p^{\prime }}$	40	40	-
$N_{pi}$, $N_{p^{\prime }i^{\prime }}$	160	160	-
$N_{ii}$, $N_{i^{\prime }i^{\prime }}$	40	40	-
$N_{pp^{\prime }}$, $N_{p^{\prime }p}$	8	8	-
$N_{ip^{\prime }}$, $N_{i^{\prime }p}$	2	2	-
$\gamma_{p}$, $\gamma_{p^{\prime }}$	$70\times 10^{-3}$	$70\times 10^{-3}$	${\mathrm{ms}}^{-1}$
$\gamma_{i}$, $\gamma_{i^{\prime }}$	$58.6\times 10^{-3}$	$58.6\times 10^{-3}$	${\mathrm{ms}}^{-1}$
${\bar{g}}_{\mathrm{AMPA}}^{\;p}$, ${\bar{g}}_{\mathrm{AMPA}}^{\;p^{\prime }}$	1	2	ms
${\bar{g}}_{\mathrm{AMPA}}^{\;i}$, ${\bar{g}}_{\mathrm{AMPA}}^{\;i^{\prime }}$	1	2	ms
${\bar{g}}_{\mathrm{GABA}}^{\;p}$, ${\bar{g}}_{\mathrm{GABA}}^{\;p^{\prime }}$	1.082	2.446	ms
${\bar{g}}_{\mathrm{GABA}}^{\;i}$, ${\bar{g}}_{\mathrm{GABA}}^{\;i^{\prime }}$	1.066	2.445	ms
$E_{\mathrm{AMPA}}$	0	0	mV
$E_{\mathrm{GABA}}$	−70	−70	mV
$E_{L}^{p}$,$E_{L}^{p^{\prime }}$	−66	−66	mV
$E_{L}^{i}$, $E_{L}^{i^{\prime }}$	−64	−64	mV
${\bar{g}}_{\mathrm{KNa}}$	1.9	1.9	$\mathrm{mS}/{\mathrm{cm}}^{2}$
$E_{K}$	−100	−100	mV
$\tau_{\mathrm{Na}}$	1.7	1.7	ms
$\alpha_{\mathrm{Na}}$	2	2	mM·ms
$R_{\mathrm{pump}}$	0.09	0.09	mM
${\mathrm{Na}}_{\mathrm{eq}}$	9.5	9.5	mM
β	1	1	-

**Table 4:** Average GABAergic conductance values for the two-cortical-column model where ${\bar{g}}_{\mathrm{AMPA}}^{\;p,\;i,\;p^{\prime },\;i^{\prime }}=2$.

Symbol	β=1	2	3	4	5	Unit
${\bar{g}}_{\mathrm{GABA}}^{\;p}$, ${\bar{g}}_{\mathrm{GABA}}^{\;p^{\prime }}$	2.446	2.599	2.752	2.905	3.058	ms
${\bar{g}}_{\mathrm{GABA}}^{\;i}$, ${\bar{g}}_{\mathrm{GABA}}^{\;i^{\prime }}$	2.445	2.576	2.708	2.84	2.971	ms

**Table 5:** Average GABAergic conductance values for the two-cortical-column model where ${\bar{g}}_{\mathrm{AMPA}}^{\;p,\;i,\;p^{\prime },\;i^{\prime }}=6$.

Symbol	β=1	2	3	4	5	Unit
${\bar{g}}_{\mathrm{GABA}}^{\;p}$, ${\bar{g}}_{\mathrm{GABA}}^{\;p^{\prime }}$	8.869	9.327	9.786	10.245	10.704	ms
${\bar{g}}_{\mathrm{GABA}}^{\;i}$, ${\bar{g}}_{\mathrm{GABA}}^{\;i^{\prime }}$	7.972	8.367	8.761	9.156	9.551	ms

**Table 6:** Average GABAergic conductance values for the two-cortical-column model where ${\bar{g}}_{\mathrm{AMPA}}^{\;p,\;i,\;p^{\prime },\;i^{\prime }}=10$.

Symbol	β=1	2	3	4	5	Unit
${\bar{g}}_{\mathrm{GABA}}^{\;p}$, ${\bar{g}}_{\mathrm{GABA}}^{\;p^{\prime }}$	15.291	16.055	16.82	17.585	18.349	ms
${\bar{g}}_{\mathrm{GABA}}^{\;i}$, ${\bar{g}}_{\mathrm{GABA}}^{\;i^{\prime }}$	13.499	14.157	14.815	15.473	16.131	ms

## Data Availability

We do not report any data. The simulation routine of the model is available at github [48].

## Supplementary Materials

The following are available at https://www.mdpi.com/article/10.3390/biology10100945/s1.

## Abbreviations

The following abbreviations are used in this manuscript:

NREM sleep	Non-rapid eye movement sleep
SWA	Slow wave activity
TMS	Transcranial magnetic stimulation
SHY	Synaptic homeostasis hypothesis
LFP	Local field potential
SWC	Sleep-waking cycle
STFT	Short-Time Fourier Transform
PSD	Power spectral density
SNR	Signal to noise ratio

## Appendix A. Neural Mass Model Equations

In this section, we provide the full model equations for the one- and two-cortical-column models. The cortical column model is described by the neural mass model of pyramidal and inhibitory populations mutually connected through AMPAergic and GABAergic synapses.

### Appendix A.1. Synapse Dynamics

Assuming that the postsynaptic response of a synapse $s_{kk^{\prime }}$—given a presynaptic population $k^{\prime }$ and a postsynaptic population *k*—is obtained by the convolution of the input at time *t* at the synaptic site H(t) with the average synaptic response to a single spike $\alpha_{k^{\prime }}$, $s_{kk^{\prime }}\left(t\right)$ takes the following form:

$$\begin{array}{cc} s_{kk^{\prime }}\left(t\right) & =H\left(t\right)\;⊛\;\alpha_{k^{\prime }}\left(t\right),\\ \; & =\int_{0}^{t}\;H\left(\tau \right)\;\alpha_{k^{\prime }}\left(t-\tau \right)\;d\tau , \end{array}$$

where $\alpha_{k^{\prime }}\left(t\right)$ has an exponential decay time course:

$$\alpha_{k^{\prime }}\left(t\right)=\gamma_{k^{\prime }}^{2}\;t\;\exp\left(-\gamma_{k^{\prime }}t\right),$$

Therefore, the first derivative of the $s_{kk^{\prime }}\left(t\right)$ with respect to *t* taking into account the Leibniz integral rule is as follows:

$$\begin{array}{cc} {\dot{s}}_{kk^{\prime }}\left(t\right) & =\frac{d}{dt}\left[\int_{0}^{t}\;H\left(\tau \right)\;\alpha_{k^{\prime }}\left(t-\tau \right)\;d\tau \right],\\ \; & =H\left(t\right)\;\alpha_{k^{\prime }}\left(t-t\right)\;+\;\int_{0}^{t}\;H\left(\tau \right)\;\frac{d}{dt}\left[\alpha_{k^{\prime }}\left(t-\tau \right)\right]\;d\tau ,\\ \; & =\int_{0}^{t}\;H\left(\tau \right)\;\left[\gamma_{k^{\prime }}^{2}\;\exp\left(-\gamma_{k^{\prime }}\left(t-\tau \right)\right)\;-\;\gamma_{k^{\prime }}^{3}\left(t-\tau \right)\;\exp\left(-\gamma_{k^{\prime }}\left(t-\tau \right)\right)\right]\;d\tau ,\\ \; & =\int_{0}^{t}\;H\left(\tau \right)\;\gamma_{k^{\prime }}^{2}\;\exp\left(-\gamma_{k^{\prime }}\left(t-\tau \right)\right)\;d\tau \;-\;\gamma_{k^{\prime }}\int_{0}^{t}\;H\left(\tau \right)\;\gamma_{k^{\prime }}^{2}\left(t-\tau \right)\;\exp\left(-\gamma_{k^{\prime }}\left(t-\tau \right)\right)\;d\tau ,\\ \; & =\int_{0}^{t}\;H\left(\tau \right)\;\gamma_{k^{\prime }}^{2}\;\exp\left(-\gamma_{k^{\prime }}\left(t-\tau \right)\right)\;d\tau \;-\;\gamma_{k^{\prime }}\int_{0}^{t}\;H\left(\tau \right)\;\alpha_{k^{\prime }}\left(t-\tau \right)\;d\tau ,\\ \; & =\int_{0}^{t}\;H\left(\tau \right)\;\gamma_{k^{\prime }}^{2}\;\exp\left(-\gamma_{k^{\prime }}\left(t-\tau \right)\right)\;d\tau \;-\;\gamma_{k^{\prime }}\;s_{kk^{\prime }}\left(t\right). \end{array}$$

In the same way, the second derivative of the $s_{kk^{\prime }}\left(t\right)$ with respect to *t* is:

$$\begin{array}{cc} {\ddot{s}}_{kk^{\prime }}\left(t\right) & =\gamma_{k^{\prime }}^{2}\;H\left(t\right)\;+\;\gamma_{k^{\prime }}\int_{0}^{t}\;H\left(\tau \right)\;\gamma_{k^{\prime }}^{2}\left(t-\tau \right)\;\exp\left(-\gamma_{k^{\prime }}\left(t-\tau \right)\right)\;d\tau \;-\;\gamma_{k^{\prime }}\;{\dot{s}}_{kk^{\prime }}\left(t\right),\\ \; & =\gamma_{k^{\prime }}^{2}\;H\left(t\right)\;-\;\gamma_{k^{\prime }}\;\left({\dot{s}}_{kk^{\prime }}\left(t\right)\;+\;\gamma_{k^{\prime }}\;s_{kk^{\prime }}\left(t\right)\right)\;-\;\gamma_{k^{\prime }}\;{\dot{s}}_{kk^{\prime }}\left(t\right),\\ \; & =\gamma_{k^{\prime }}^{2}\;H\left(t\right)\;-\;\gamma_{k^{\prime }}^{2}\;s_{kk^{\prime }}\left(t\right)\;-\;2\gamma_{k^{\prime }}\;{\dot{s}}_{kk^{\prime }}\left(t\right),\\ \; & =\gamma_{k^{\prime }}^{2}\;\left[H\left(t\right)\;-\;s_{kk^{\prime }}\left(t\right)\right]\;-\;2\gamma_{k^{\prime }}\;{\dot{s}}_{kk^{\prime }}\left(t\right), \end{array}$$

Finally, by substituting the $H\left(t\right)=N_{kk^{\prime }}\;Q_{k^{\prime }}\left(V_{k^{\prime }}\right)+\phi$—which is the presynaptic drive at synapse of population *k*—the postsynaptic response $s_{kk^{\prime }}$ takes the form of:

$${\ddot{s}}_{kk^{\prime }}=\gamma_{k^{\prime }}^{2}\;\left(N_{kk^{\prime }}\;Q_{k^{\prime }}\left(V_{k^{\prime }}\right)+\phi -s_{kk^{\prime }}\right)\;-2\gamma_{k^{\prime }},\;{\dot{s}}_{kk^{\prime }}$$

Please note that the noise term ϕ is zero for the inhibitory synapses and is a stochastic Gaussian process with zero autocorrelation time constant for the excitatory synapses.

### Appendix A.2. One-Cortical-Column Model

The one-cortical-column model consists of one pyramidal and one inhibitory population (see panel A in Figure 1). The model utilizes the mathematical formalism of the Hodgkin–Huxley model to describe the membrane voltage activity in terms of driving synaptic activity. The synaptic activity contains one leak, two synaptic (AMPAergic and GABAergic) currents and one activity-dependent potassium current. In the absence of extrinsic input (the Gaussian process), the system settles down on the steady state solution. At every time point, the extrinsic input pushes the system out of the steady state solution, however, the aforementioned synaptic currents drive the system back to the steady state solution. The full model equations for the one-cortical-column model are described by:

$$\begin{array}{cc} \tau_{p}{\dot{V}}_{p} & =-I_{L}^{p}-I_{\mathrm{AMPA}}^{p}-I_{\mathrm{GABA}}^{p}-\tau_{p}C_{m}^{-1}I_{\mathrm{KNa}},\\ \tau_{i}\dot{V_{i}} & =-I_{L}^{i}-I_{\mathrm{AMPA}}^{i}-I_{\mathrm{GABA}}^{i},\\ {\ddot{s}}_{pp} & =\gamma_{p}^{2}\;\left(N_{pp}\;Q_{p}\left(V_{p}\right)+\phi -s_{pp}\right)\;-2\gamma_{p}\;{\dot{s}}_{pp},\\ {\ddot{s}}_{pi} & =\gamma_{i}^{2}\;\left(N_{pi}\;Q_{i}\left(V_{i}\right)-s_{pi}\right)\;-2\gamma_{i}\;{\dot{s}}_{pi},\\ {\ddot{s}}_{ip} & =\gamma_{p}^{2}\;\left(N_{ip}\;Q_{p}\left(V_{p}\right)+\phi -s_{ip}\right)\;-2\gamma_{p}\;{\dot{s}}_{ip},\\ {\ddot{s}}_{ii} & =\gamma_{i}^{2}\;\left(N_{ii}\;Q_{i}\left(V_{i}\right)-s_{ii}\right)\;-2\gamma_{i}\;{\dot{s}}_{ii},\\ \tau_{\mathrm{Na}}\;\dot{\left[\mathrm{Na}\right]} & =\alpha_{\mathrm{Na}}Q_{p}\left(V_{p}\right)-{\mathrm{Na}}_{\mathrm{pump}}\left(\left[\mathrm{Na}\right]\right), \end{array}$$

with the currents defined by:

$$\begin{array}{cc} I_{L}^{k} & ={\bar{g}}_{L}\left(V_{k}-E_{L}^{k}\right),\\ I_{\mathrm{AMPA}}^{k} & ={\bar{g}}_{\mathrm{AMPA}}^{\;k}s_{kp}\left(V_{k}-E_{\mathrm{AMPA}}\right),\\ I_{\mathrm{GABA}}^{k} & ={\bar{g}}_{\mathrm{GABA}}^{\;k}s_{ki}\left(V_{k}-E_{\mathrm{GABA}}\right),\\ I_{\mathrm{KNa}} & ={\bar{g}}_{\mathrm{KNa}}\;\frac{0.37}{1+{\left(\frac{38.7}{\left[\mathrm{Na}\right]}\right)}^{3.5}}\;\left(V_{p}-E_{K}\right), \end{array}$$

the sodium pump and the cortical firing rate functions are given by:

$$\begin{array}{cc} {\mathrm{Na}}_{\mathrm{pump}}\left(\left[\mathrm{Na}\right]\right) & =R_{\mathrm{pump}}\left(\frac{{\left[\mathrm{Na}\right]}^{3}}{{\left[\mathrm{Na}\right]}^{3}+3375}-\frac{{\left[\mathrm{Na}\right]}_{\mathrm{eq}}^{3}}{{\left[\mathrm{Na}\right]}_{\mathrm{eq}}^{3}+3375}\right),\\ Q_{k}\left(V_{k}\right) & =Q_{k}^{max}\frac{\left(1+\tanh\left(C\left(V_{k}-\theta_{k}\right)/\sigma_{k}\right)\right)}{2},\;C=\frac{\pi }{2\sqrt{3}} \end{array}$$

Symbol descriptions and the parameter values are provided in Table 1 and Table 2, respectively.

### Appendix A.3. Two-Cortical-Column Model

The two-cortical-column model consists of two cortical columns each containing one pyramidal and one inhibitory population (see Appendix A.2) that are mutually coupled through AMPAergic connections (see panel B in Figure 1). The full model equations for the two-cortical-column model are described by:

$$\begin{array}{cc} \tau_{p}{\dot{V}}_{p} & =-I_{L}^{p}-I_{\mathrm{AMPA}}^{p}-I_{\mathrm{GABA}}^{p}-\tau_{p}C_{m}^{-1}I_{\mathrm{KNa}}^{p},\\ \tau_{i}\dot{V_{i}} & =-I_{L}^{i}-I_{\mathrm{AMPA}}^{i}-I_{\mathrm{GABA}}^{i},\\ \tau_{p^{\prime }}{\dot{V}}_{p^{\prime }} & =-I_{L}^{p^{\prime }}-I_{\mathrm{AMPA}}^{p^{\prime }}-I_{\mathrm{GABA}}^{p^{\prime }}-\tau_{p^{\prime }}C_{m}^{-1}I_{\mathrm{KNa}}^{p^{\prime }},\\ \tau_{i^{\prime }}\dot{V_{i^{\prime }}} & =-I_{L}^{i^{\prime }}-I_{\mathrm{AMPA}}^{i^{\prime }}-I_{\mathrm{GABA}}^{i^{\prime }},\\ {\ddot{s}}_{pp} & =\gamma_{p}^{2}\;\left(N_{pp}\;Q_{p}\left(V_{p}\right)+\phi -s_{pp}\right)\;-2\gamma_{p}\;{\dot{s}}_{pp},\\ {\ddot{s}}_{pi} & =\gamma_{i}^{2}\;\left(N_{pi}\;Q_{i}\left(V_{i}\right)-s_{pi}\right)\;-2\gamma_{i}\;{\dot{s}}_{pi},\\ {\ddot{s}}_{ip} & =\gamma_{p}^{2}\;\left(N_{ip}\;Q_{p}\left(V_{p}\right)+\phi -s_{ip}\right)\;-2\gamma_{p}\;{\dot{s}}_{ip},\\ {\ddot{s}}_{ii} & =\gamma_{i}^{2}\;\left(N_{ii}\;Q_{i}\left(V_{i}\right)-s_{ii}\right)\;-2\gamma_{i}\;{\dot{s}}_{ii},\\ {\ddot{s}}_{p^{\prime }p^{\prime }} & =\gamma_{p^{\prime }}^{2}\;\left(N_{p^{\prime }p^{\prime }}\;Q_{p^{\prime }}\left(V_{p^{\prime }}\right)+\phi -s_{p^{\prime }p^{\prime }}\right)\;-2\gamma_{p^{\prime }}\;{\dot{s}}_{p^{\prime }p^{\prime }},\\ {\ddot{s}}_{p^{\prime }i^{\prime }} & =\gamma_{i^{\prime }}^{2}\;\left(N_{p^{\prime }i^{\prime }}\;Q_{i^{\prime }}\left(V_{i^{\prime }}\right)-s_{p^{\prime }i^{\prime }}\right)\;-2\gamma_{i^{\prime }}\;{\dot{s}}_{p^{\prime }i^{\prime }},\\ {\ddot{s}}_{i^{\prime }p^{\prime }} & =\gamma_{p^{\prime }}^{2}\;\left(N_{i^{\prime }p^{\prime }}\;Q_{p^{\prime }}\left(V_{p^{\prime }}\right)+\phi -s_{i^{\prime }p^{\prime }}\right)\;-2\gamma_{p^{\prime }}\;{\dot{s}}_{i^{\prime }p^{\prime }},\\ {\ddot{s}}_{i^{\prime }i^{\prime }} & =\gamma_{i^{\prime }}^{2}\;\left(N_{i^{\prime }i^{\prime }}\;Q_{i^{\prime }}\left(V_{i^{\prime }}\right)-s_{i^{\prime }i^{\prime }}\right)\;-2\gamma_{i^{\prime }}\;{\dot{s}}_{i^{\prime }i^{\prime }},\\ {\ddot{s}}_{pp^{\prime }}^{\;\mathrm{inter}} & =\gamma_{p^{\prime }}^{2}\;\left(N_{pp^{\prime }}\;Q_{p^{\prime }}\left(V_{p^{\prime }}\right)-s_{pp^{\prime }}^{\;\mathrm{inter}}\right)\;-2\gamma_{p^{\prime }}\;{\dot{s}}_{pp^{\prime }}^{\;\mathrm{inter}},\\ {\ddot{s}}_{ip^{\prime }}^{\;\mathrm{inter}} & =\gamma_{p^{\prime }}^{2}\;\left(N_{ip^{\prime }}\;Q_{p^{\prime }}\left(V_{p^{\prime }}\right)-s_{ip^{\prime }}^{\;\mathrm{inter}}\right)\;-2\gamma_{p^{\prime }}\;{\dot{s}}_{ip^{\prime }}^{\;\mathrm{inter}},\\ {\ddot{s}}_{p^{\prime }p}^{\;\mathrm{inter}} & =\gamma_{p}^{2}\;\left(N_{p^{\prime }p}\;Q_{p}\left(V_{p}\right)-s_{p^{\prime }p}^{\;\mathrm{inter}}\right)\;-2\gamma_{p}\;{\dot{s}}_{p^{\prime }p}^{\;\mathrm{inter}},\\ {\ddot{s}}_{i^{\prime }p}^{\;\mathrm{inter}} & =\gamma_{p}^{2}\;\left(N_{i^{\prime }p}\;Q_{p}\left(V_{p}\right)-s_{i^{\prime }p}^{\;\mathrm{inter}}\right)\;-2\gamma_{p}\;{\dot{s}}_{i^{\prime }p}^{\;\mathrm{inter}},\\ \tau_{\mathrm{Na}}\;{\dot{\left[\mathrm{Na}\right]}}_{p} & =\alpha_{\mathrm{Na}}Q_{p}\left(V_{p}\right)-{\mathrm{Na}}_{\mathrm{pump}}\left({\left[\mathrm{Na}\right]}_{p}\right),\\ \tau_{\mathrm{Na}}\;{\dot{\left[\mathrm{Na}\right]}}_{p^{\prime }} & =\alpha_{\mathrm{Na}}Q_{p^{\prime }}\left(V_{p^{\prime }}\right)-{\mathrm{Na}}_{\mathrm{pump}}\left({\left[\mathrm{Na}\right]}_{p^{\prime }}\right), \end{array}$$

where $I_{\mathrm{KNa}}^{h}$ is the sodium-dependent potassium current of either the perturbed pyramidal population (h=p) or the unperturbed pyramidal population ($h=p^{\prime }$). The currents are defined by:

$$\begin{array}{cc} I_{L}^{k} & ={\bar{g}}_{L}\left(V_{k}-E_{L}^{k}\right),\\ I_{\mathrm{AMPA}}^{k} & ={\bar{g}}_{\mathrm{AMPA}}^{\;k}\;s_{k(p/p^{\prime })}\;\left(V_{k}-E_{\mathrm{AMPA}}\right)\;+\;\beta \cdot {\bar{g}}_{\mathrm{AMPA}}^{\;k}\;s_{k(p^{\prime }/p)}^{\;\mathrm{inter}}\;\left(V_{k}-E_{\mathrm{AMPA}}\right),\\ I_{\mathrm{GABA}}^{k} & ={\bar{g}}_{\mathrm{GABA}}^{\;k}s_{k(i/i^{\prime })}\left(V_{k}-E_{\mathrm{GABA}}\right),\\ I_{\mathrm{KNa}}^{h} & ={\bar{g}}_{\mathrm{KNa}}\;\frac{0.37}{1+{\left(\frac{38.7}{{\left[\mathrm{Na}\right]}_{h}}\right)}^{3.5}}\;\left(V_{h}-E_{K}\right), \end{array}$$

the sodium pump and the cortical firing rate functions are given by:

$$\begin{array}{cc} {\mathrm{Na}}_{\mathrm{pump}}\left({\left[\mathrm{Na}\right]}_{h}\right) & =R_{\mathrm{pump}}\left(\frac{{\left[\mathrm{Na}\right]}_{h}^{3}}{{\left[\mathrm{Na}\right]}_{h}^{3}+3375}-\frac{{\left[\mathrm{Na}\right]}_{\mathrm{eq}}^{3}}{{\left[\mathrm{Na}\right]}_{\mathrm{eq}}^{3}+3375}\right),\\ Q_{k}\left(V_{k}\right) & =Q_{k}^{max}\frac{\left(1+\tanh\left(C\left(V_{k}-\theta_{k}\right)/\sigma_{k}\right)\right)}{2},\;C=\frac{\pi }{2\sqrt{3}} \end{array}$$

Symbol descriptions and the parameter values are provided in Table 1 and Table 3, respectively.

## Appendix B. Dynamical Constrains on $\bar{g}$ GABA k

### Appendix B.1. One-Cortical-Column Model

Upscaling the conductance of excitatory synapses by increasing the average AMPAergic conductance, ${\bar{g}}_{\mathrm{AMPA}}^{\;k}$, which is in agreement with SHY allows us to place the model from NREM sleep dynamics into wakefulness. The average GABAergic conductance on pyramidal and inhibitory population are increased to counterbalance the more excitation each of the populations receives due to upscaling the average AMPAergic conductance. To do so, we use the peaks of $V_{p}$ and $V_{i}$ values during Up states (see Section 2.8.2 for Up and Down definition) as the steady state value of the average membrane potential of pyramidal and inhibitory populations during wakefulness. The average GABAergic conductance on pyramidal and inhibitory populations are increased until these $V_{p}$ and $V_{i}$ values are obtained for the ${\bar{g}}_{\mathrm{AMPA}}^{\;k}=2\;$ responsible of wakefulness.

The steady state solution of the model equations for the one-cortical-column (see Appendix A.2) are obtained by setting derivative of all variables to zero.

$$\begin{array}{cc} 0 & =-I_{L}^{p}-I_{\mathrm{AMPA}}^{p}-I_{\mathrm{GABA}}^{p}-\tau_{p}C_{m}^{-1}I_{\mathrm{KNa}}^{p},\\ 0 & =-I_{L}^{i}-I_{\mathrm{AMPA}}^{i}-I_{\mathrm{GABA}}^{i},\\ s_{kk^{\prime }} & =N_{kk^{\prime }}\;Q_{k^{\prime }}\left(V_{k^{\prime }}\right),\\ \left[\mathrm{Na}\right] & =\sqrt[{3}]{\frac{A\cdot 3375}{1-A}},\\ A & =\frac{\alpha_{\mathrm{Na}}}{R_{\mathrm{pump}}}Q_{p}\left(V_{p}\right)+\frac{{\left[\mathrm{Na}\right]}_{\mathrm{eq}}^{3}}{{\left[\mathrm{Na}\right]}_{\mathrm{eq}}^{3}+3375}, \end{array}$$

therefore, the average GABAergic conductance on pyramidal and inhibitory populations, which is required to keep the average membrane potential of pyramidal and inhibitory populations during wakefulness equal to the peaks of $V_{p}$ and $V_{i}$ during Up states, is as follows:

$$\begin{array}{cc} {\bar{g}}_{\mathrm{GABA}}^{\;p} & =\frac{-{\bar{g}}_{L}\left(V_{p}-E_{L}^{p}\right)-{\bar{g}}_{\mathrm{AMPA}}^{\;p}s_{pp}\left(V_{p}-E_{\mathrm{AMPA}}\right)-{\bar{g}}_{\mathrm{KNa}}\;\frac{0.37}{1+{\left(\frac{38.7}{\left[\mathrm{Na}\right]}\right)}^{3.5}}\;\left(V_{p}-E_{K}\right)}{s_{pi}\left(V_{p}-E_{\mathrm{GABA}}\right)},\\ {\bar{g}}_{\mathrm{GABA}}^{\;i} & =\frac{-{\bar{g}}_{L}\left(V_{p}-E_{L}^{i}\right)-{\bar{g}}_{\mathrm{AMPA}}^{\;i}s_{ip}\left(V_{p}-E_{\mathrm{AMPA}}\right)}{s_{ii}\left(V_{i}-E_{\mathrm{GABA}}\right)}, \end{array}$$

The average GABAergic conductance of the pyramidal population and that of the inhibitory population during wakefulness are obtained by setting ${\bar{g}}_{\mathrm{AMPA}}^{\;p/i}=2$ and the peaks of $V_{p}$ and $V_{i}$ values during Up states in the above equations.

### Appendix B.2. Two-Cortical-Column Model

Inter-cortical column coupling introduces more excitation to the pyramidal and inhibitory populations. To keep the steady state value of $V_{p}$ and $V_{i}$ in the two-cortical-column scenario equal to the ones in the one-cortical-column model both during NREM sleep and wakefulness, the average GABAergic conductances on pyramidal and inhibitory populations are increased to counterbalance this inter-cortical column coupling.

The steady state solution of the model equations for the two-cortical-column (see Appendix A.3) is obtained by setting the derivatives of all variables to zero:

$$\begin{array}{cc} 0 & =-I_{L}^{p/p^{\prime }}-I_{\mathrm{AMPA}}^{p/p^{\prime }}-I_{\mathrm{GABA}}^{p/p^{\prime }}-\tau_{p/p^{\prime }}C_{m}^{-1}I_{\mathrm{KNa}}^{p/p^{\prime }},\\ 0 & =-I_{L}^{i/i^{\prime }}-I_{\mathrm{AMPA}}^{i/i^{\prime }}-I_{\mathrm{GABA}}^{i/i^{\prime }},\\ s_{kk^{\prime }} & =N_{kk^{\prime }}\;Q_{k^{\prime }}\left(V_{k^{\prime }}\right),\\ s_{kk^{\prime }}^{\;\mathrm{inter}} & =N_{kk^{\prime }}\;Q_{k^{\prime }}\left(V_{k^{\prime }}\right)\\ {\left[\mathrm{Na}\right]}_{p/p^{\prime }} & =\sqrt[{3}]{\frac{A_{p/p^{\prime }}\cdot 3375}{1-A_{p/p^{\prime }}}},\\ A_{p/p^{\prime }} & =\frac{\alpha_{\mathrm{Na}}}{R_{\mathrm{pump}}}Q_{p/p^{\prime }}\left(V_{p/p^{\prime }}\right)+\frac{{\left[\mathrm{Na}\right]}_{\mathrm{eq}}^{3}}{{\left[\mathrm{Na}\right]}_{\mathrm{eq}}^{3}+3375}, \end{array}$$

by taking into account the symmetry between the two cortical columns, we set the steady state value of $V_{p}$ and $V_{i}$ equal to $V_{p^{\prime }}$ and $V_{i^{\prime }}$ ($V_{p^{\prime }}=V_{p}$ and $V_{i^{\prime }}=V_{i}$), respectively. Therefore, to keep the average membrane potential of pyramidal and inhibitory populations in the two-cortical-column scenario equal to the ones in the one-cortical-column model both during NREM sleep and wakefulness, the average GABAergic conductances on pyramidal and inhibitory populations change as follows:

$$\begin{array}{cc} {\bar{g}}_{\mathrm{GABA}}^{\;p} & =\frac{-{\bar{g}}_{L}\left(V_{p}-E_{L}^{p}\right)-{\bar{g}}_{\mathrm{AMPA}}^{\;p}s_{pp}\left(V_{p}-E_{\mathrm{AMPA}}\right)-\beta \;{\bar{g}}_{\mathrm{AMPA}}^{\;p}s_{pp}\left(V_{p}-E_{\mathrm{AMPA}}\right)-{\bar{g}}_{\mathrm{KNa}}\;\frac{0.37}{1+{\left(\frac{38.7}{\left[\mathrm{Na}\right]}\right)}^{3.5}}\;\left(V_{p}-E_{K}\right)}{s_{pi}\left(V_{p}-E_{\mathrm{GABA}}\right)},\\ {\bar{g}}_{\mathrm{GABA}}^{\;i} & =\frac{-{\bar{g}}_{L}\left(V_{p}-E_{L}^{i}\right)-{\bar{g}}_{\mathrm{AMPA}}^{\;i}s_{ip}\left(V_{p}-E_{\mathrm{AMPA}}\right)-\beta \;{\bar{g}}_{\mathrm{AMPA}}^{\;i}s_{ip}\left(V_{p}-E_{\mathrm{AMPA}}\right)}{s_{ii}\left(V_{i}-E_{\mathrm{GABA}}\right)}, \end{array}$$

The average GABAergic conductances on pyramidal and inhibitory populations during NREM sleep in the two-cortical-column scenario are obtained by setting ${\bar{g}}_{\mathrm{AMPA}}^{\;p/i}=1$ and the steady state values of $V_{p}$ and $V_{i}$ during NREM sleep in the one-cortical-column scenario. Therefore, the one cortical column in NREM sleep regime is run for 15 seconds using a stochastic Heun method [47] with a step size of 0.1 ms in the absence of noise to obtain the steady state values of $V_{p}$ and $V_{i}$.

The average GABAergic conductances on pyramidal and inhibitory populations during wakefulness in the two-cortical-column scenario are obtained by setting ${\bar{g}}_{\mathrm{AMPA}}^{\;p/i}=2$, β=1 and the peaks of $V_{p}$ and $V_{i}$ values during Up states in the one-cortical-column scenario in the aforementioned equations.

Moreover, the average GABAergic conductances on pyramidal and inhibitory populations during wakefulness in the two-cortical-column scenario for variations of β and ${\bar{g}}_{\mathrm{AMPA}}^{\;k}$ are obtained by setting the corresponding β, ${\bar{g}}_{\mathrm{AMPA}}^{\;p/i}$ and the peaks of $V_{p}$ and $V_{i}$ values during Up states in the one-cortical-column scenario in the aforementioned equations.
