# Supplementary material for: Computational Modeling of Information Propagation during the Sleep–Waking Cycle
Source: Biology (Basel). 2021 Sep 22;10(10):945. doi: 10.3390/biology10100945 (PMC8533346; doi:10.3390/biology10100945)
Supplement: Supplementary file 1 [file biology-10-00945-s001.zip › biology-1334040-supplementary/biology-1334040-supplementary.pdf]

Supplementary Materials: Computational Modeling of Information Propagation During the Sleep–Waking Cycle

Farhad Razi [1,2], Rubén Moreno Bote [2] and Belén Sancristóbal [1,*]

The PDF file includes supplementary figures.

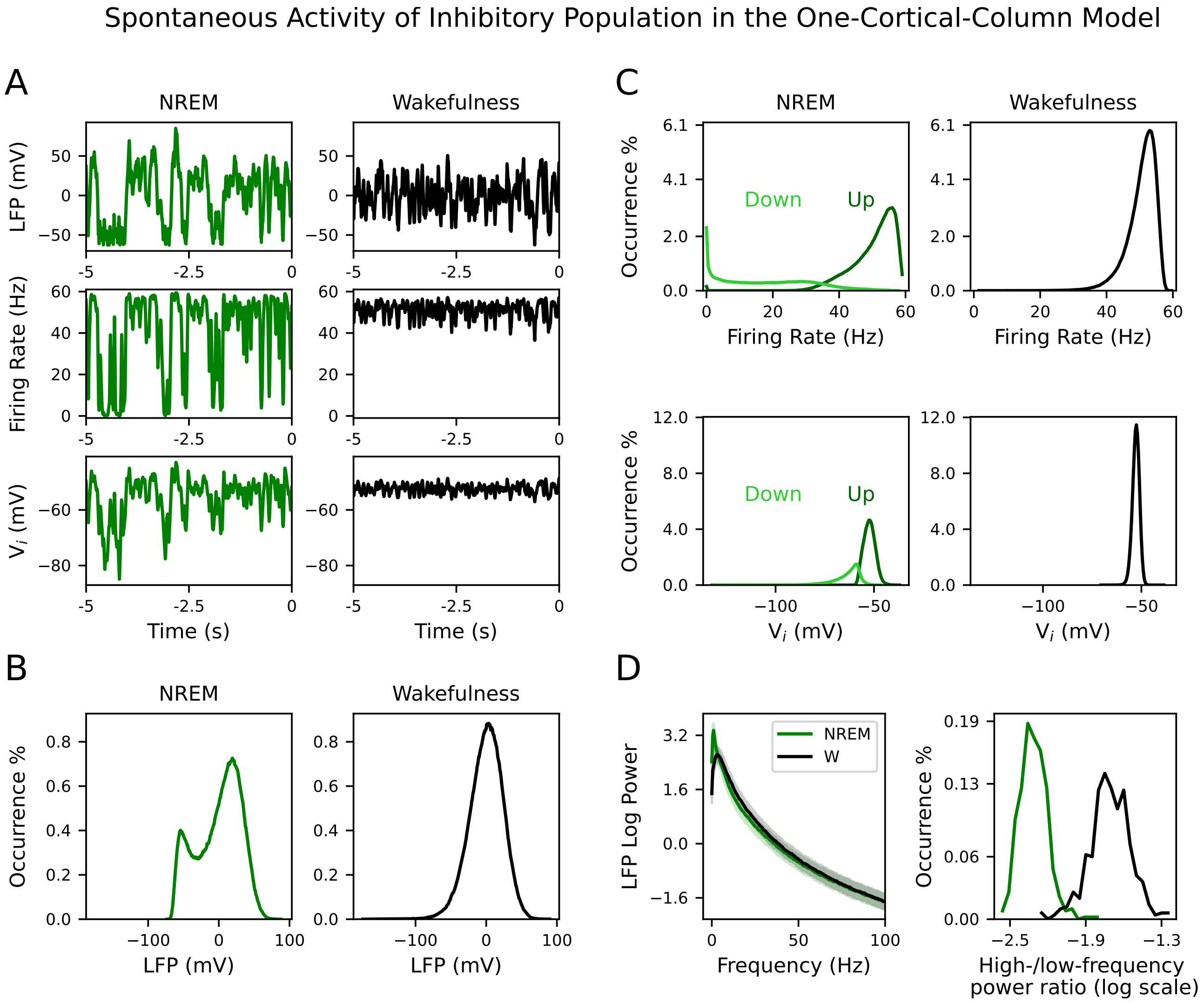

Figure S1. Spontaneous activity ($\xi = 0$) during NREM sleep and wakefulness in the one-cortical-column model as in Figure 2 but for inhibitory population. (A) Simulated signals from one random simulation as in Figure 2A. (B) Distribution of the LFP signals as in Figure 2B. (C) Top row: distribution of the firing rate during Up (dark green) and Down states (light green). Bottom row: distribution of the V_p signal during Up (dark green) and Down (light green) events as in Figure 2C. (D) Spectral content of LFP signals during NREM sleep and wakefulness. Left: average power spectrum density of LFP signals during NREM sleep (green) and wakefulness (black). Right: distribution of high-/low-frequency power ratio in the LFP during NREM sleep (green) and wakefulness (black) as in Figure 2D.

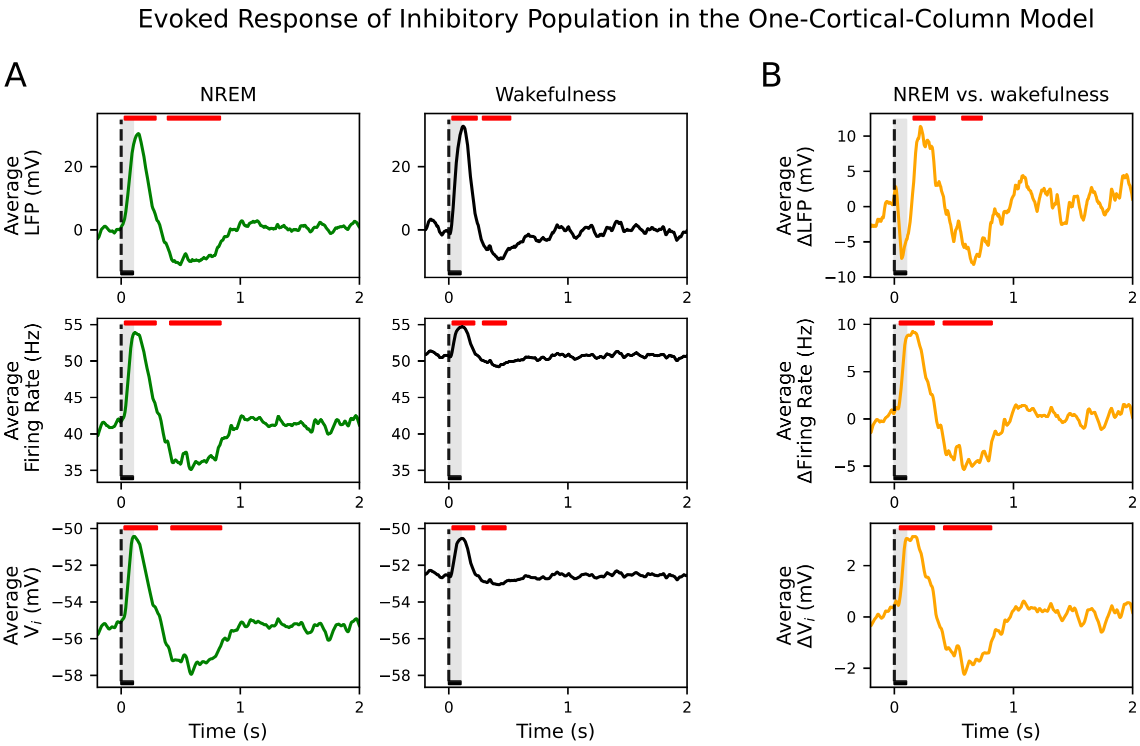

Figure S2. Evoked response in the one-cortical-column as in Figure 3 but for inhibitory population. (A) Average LFP, firing rate and V_p across 500 simulations for NREM sleep (left column) and wakefulness (right column). Red horizontal bars show the location and length of significant t-cluster statistics when comparing the poststimulus versus the prestimulus intervals. In all signals, responses to the external stimulus consist of one positive and one negative significant deflection. The positive ($P < 0.0009$) and negative ($P < 0.001$) deflections are significant in NREM and wakefulness. (B) Average difference ΔLFP, Δfiring rate and ΔV_p between NREM sleep and wakefulness across 500 simulations. Red horizontal bars show the location and length of significant t-cluster statistics when comparing the poststimulus intervals during NREM sleep with wakefulness. The positive ($P < 0.0008$) and negative ($P < 0.001$) deflections in all signals are significant. The amplitude of both deflections are higher in NREM sleep than in wakefulness in all signal types. In all cases, the dashed vertical line, aligned at zero, indicates stimulus ξ onset. Stimulus duration is represented by a black horizontal line and light gray area.

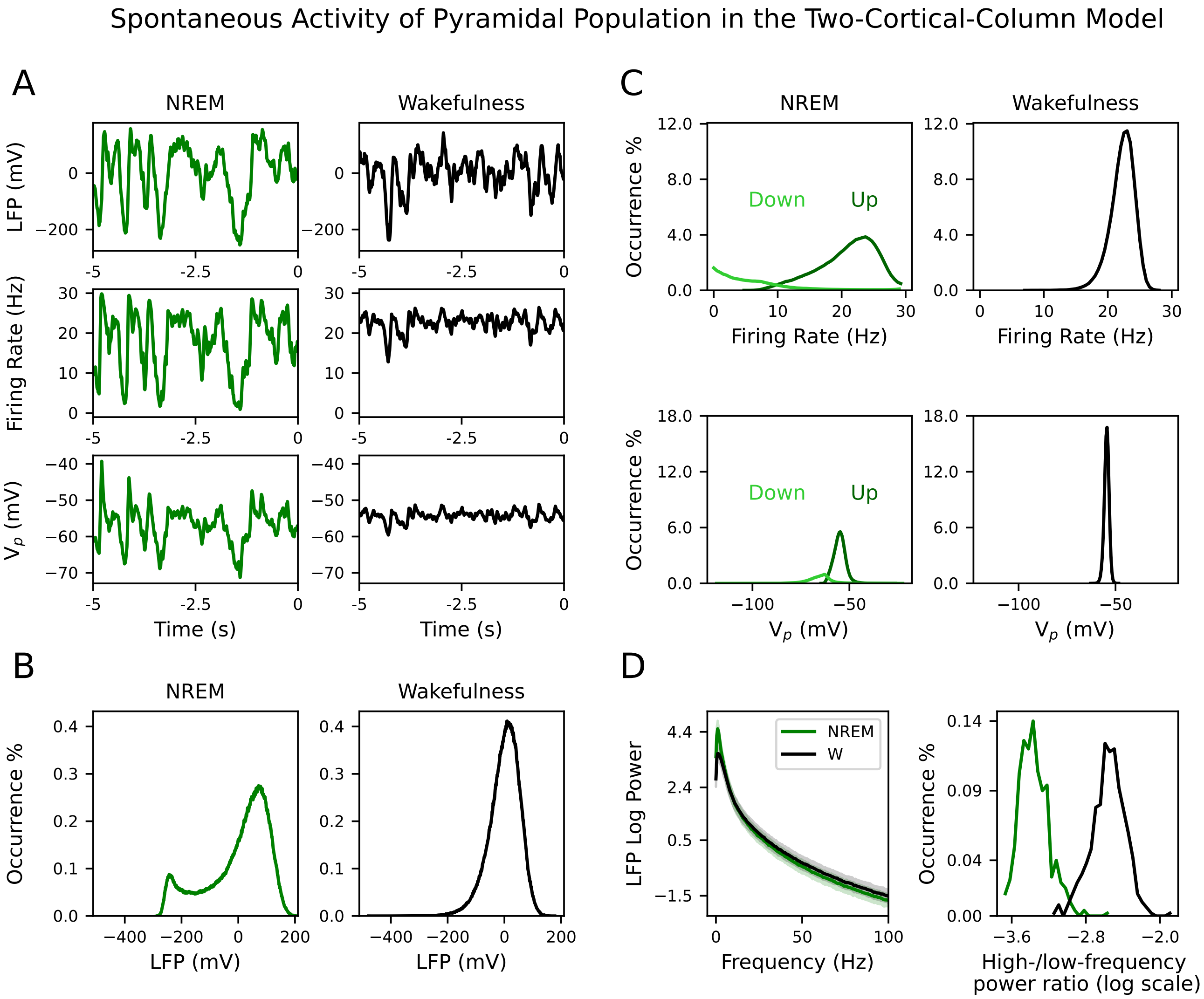

Figure S3. Spontaneous activity ($\xi = 0$) during NREM sleep and wakefulness in the two-cortical-column model. (A) Simulated signals from one random simulation as in Figure 2A. (B) Distribution of the LFP signals as in Figure 2B. (C) Top row: distribution of the firing rate during Up (dark green) and Down states (light green). Bottom row: distribution of the V_p signal during Up (dark green) and Down (light green) events as in Figure 2C. (D) Spectral content of LFP signals during NREM sleep and wakefulness. Left: average power spectrum density of LFP signals during NREM sleep (green) and wakefulness (black). Right: distribution of high-/low-frequency power ratio in the LFP during NREM sleep (green) and wakefulness (black) as in Figure 2D. Here, $\beta = 1$ and $g_{AMPA} = 2$. Note that this figure and Figure 2 are qualitatively similar.

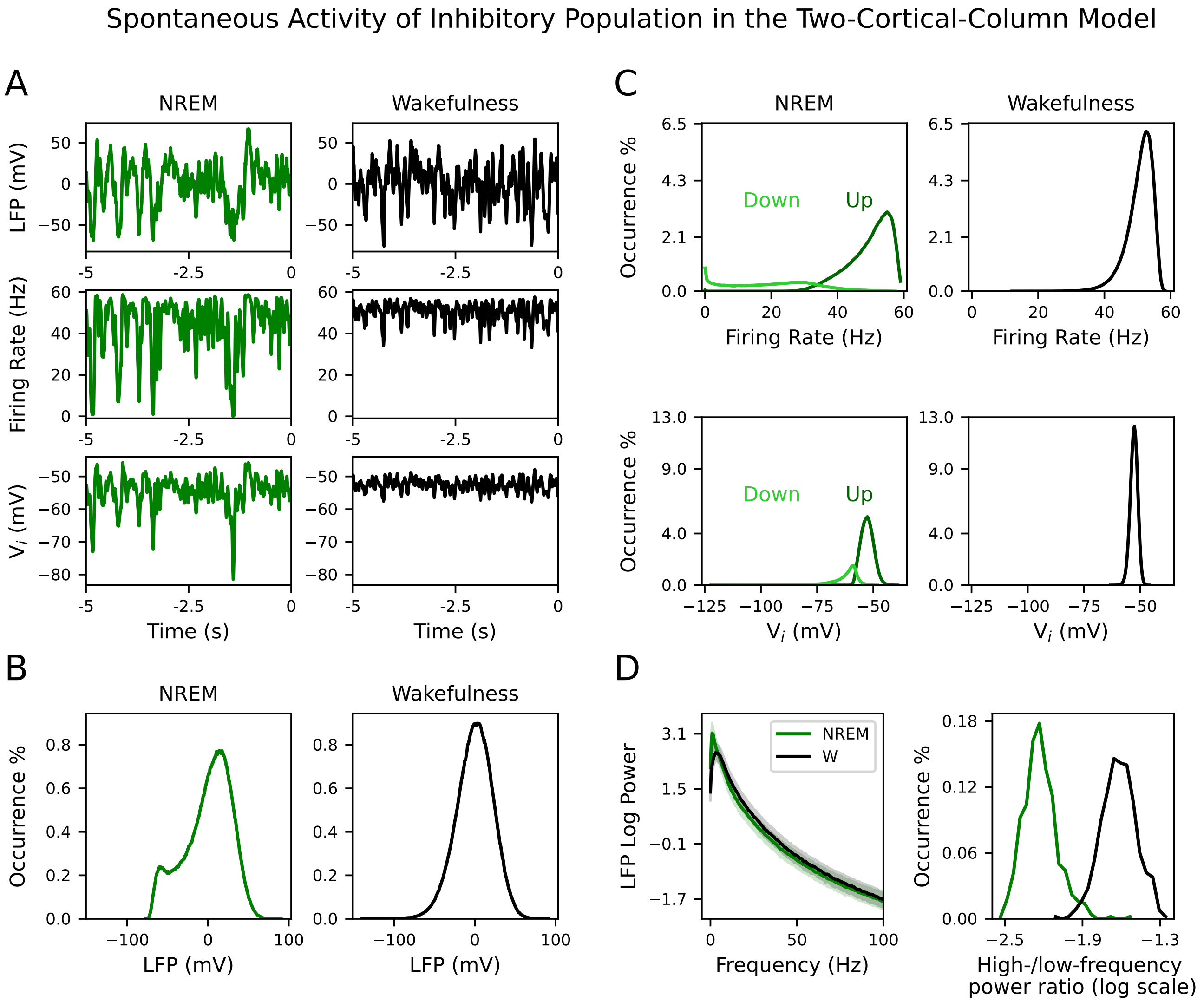

Figure S4. Spontaneous activity ($\xi = 0$) during NREM sleep and wakefulness in the two-cortical-column model as in Figure S3 but for inhibitory population. (A) Simulated signals from one random simulation as in Figure 2A. (B) Distribution of the LFP signals as in Figure 2B. (C) Top row: distribution of the firing rate during Up (dark green) and Down states (light green). Bottom row: distribution of the V_p signal during Up (dark green) and Down (light green) events as in Figure 2C. (D) Spectral content of LFP signals during NREM sleep and wakefulness. Left: average power spectrum density of LFP signals during NREM sleep (green) and wakefulness (black). Right: distribution of high-/low-frequency power ratio in the LFP during NREM sleep (green) and wakefulness (black) as in Figure 2D. Here, $\beta = 1$ and $g_{\text{AMPA}} = 2$. Note that this figure and Figure S1 are qualitatively similar.

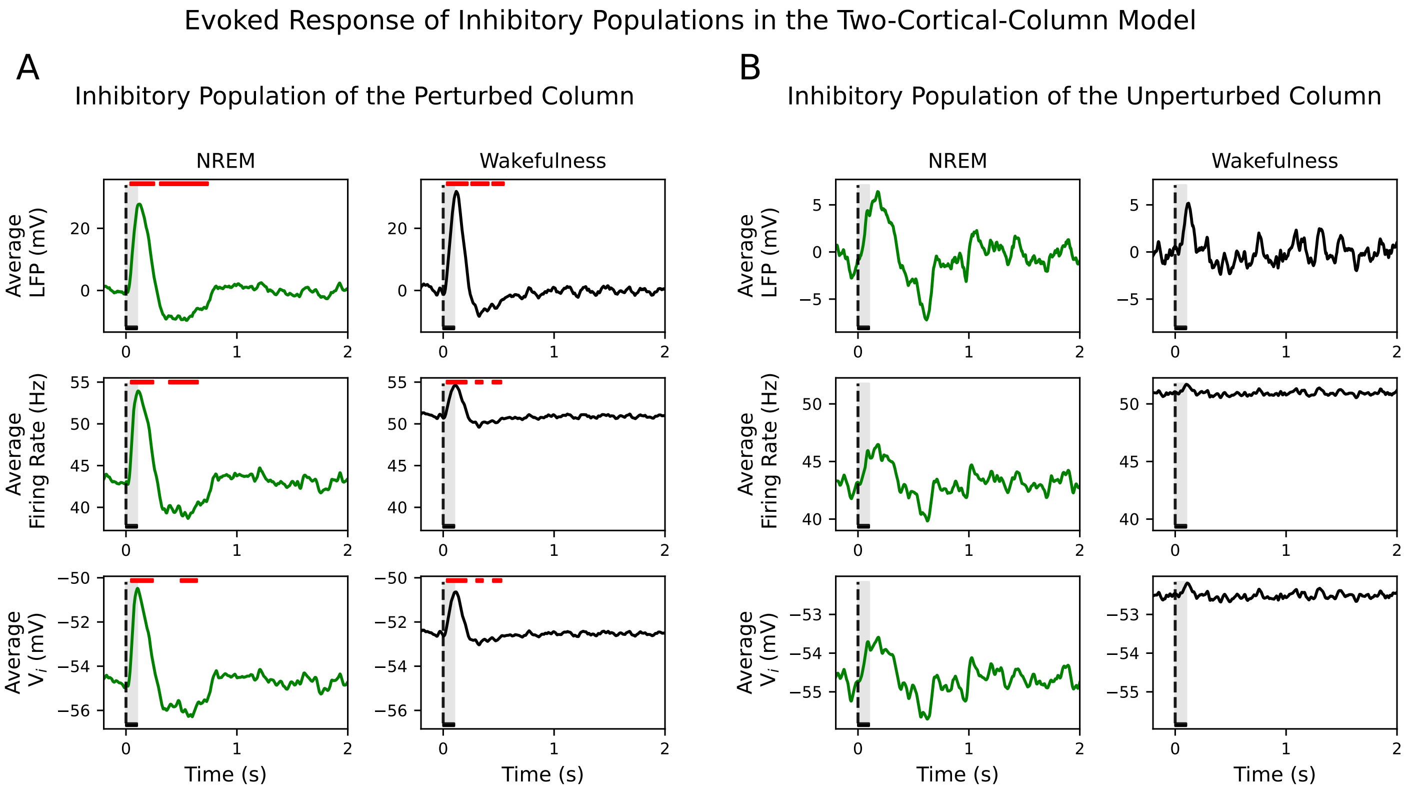

Figure S5. Evoked responses in the two-cortical-column model where $\beta = 1$ and $\bar{g}_{\text{AMPA}} = 2$ as in Figure 4 but for inhibitory population. (A) Average LFP, firing rate and V_p across 500 simulations for NREM sleep (left column) and wakefulness (right column) in the perturbed pyramidal population. Red horizontal bars show the location and length of significant t-cluster statistics when comparing the poststimulus versus the prestimulus interval. In all signals, responses to the external stimulus consist of one positive and negative significant deflection. The positive ($P < 0.0008$) and negative ($P < 0.001$) deflections are significant in NREM and wakefulness. Note that the coupling between the two-cortical-columns does not change the significance of positive and negative deflections in the evoked responses. (B) Results are as in panel (A) but for the case of the unperturbed pyramidal population. The evoked responses do not show significant deflections during NREM sleep and wakefulness in the LFP signal nor in the firing rate nor in V_p, where the increase in activity is not significantly higher than the spontaneous activity. In all cases, the dashed vertical line, aligned at zero, indicates stimulus ξ onset. Stimulus duration is represented by a black horizontal line and light gray area.

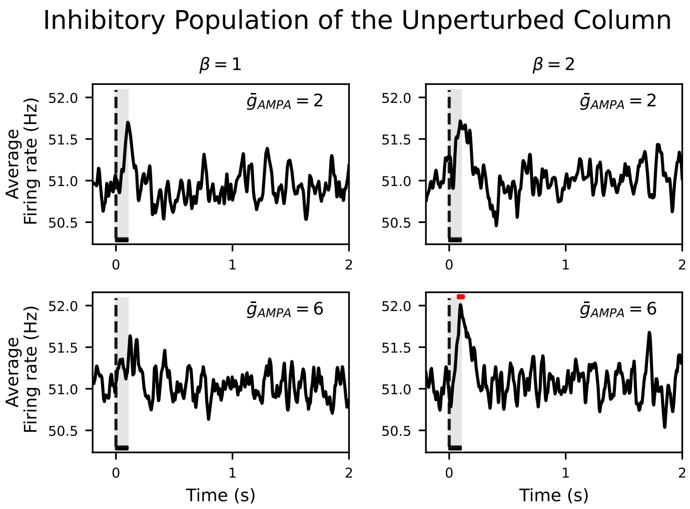

Figure S6. Evoked responses in the two-cortical-column model for $\beta = 1,\ 2$ and $\bar{g}_{\mathrm{AMPA}} = 2,\ 6$ as in Figure 5 but for inhibitory population. Average across 500 simulations of the firing rate of the unperturbed pyramidal population. Red horizontal bars show the location and length of significant t-cluster statistics when comparing the poststimulus versus the prestimulus interval. Firing rate of the unperturbed population shows a positive deflection after stimulus onset in all cases. Increasing $\bar{g}_{\mathrm{AMPA}}$ from 2 to 6 evokes a significant response ($P < 0.001$) in the unperturbed population only if β is also increased from 1 to 2. In all cases, the dashed vertical line, aligned at zero, indicates stimulus ξ onset. Stimulus duration is represented by a black horizontal line and light gray area.

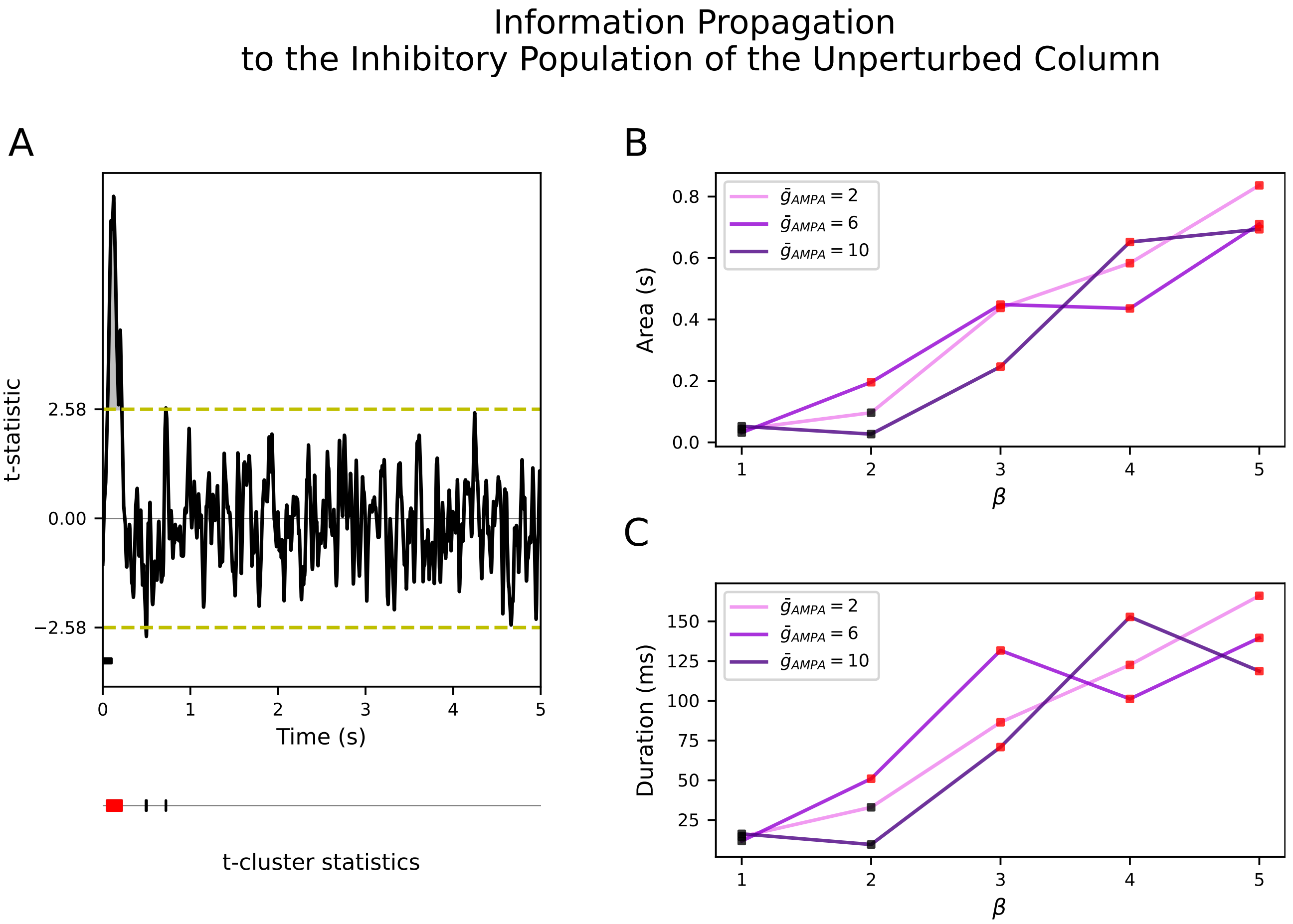

Figure S7. Information propagation as a function of β and $\bar{g}_{\text{AMPA}}$ as in Figure 6 but for inhibitory population. (A) Example of extraction of t-cluster statistics when comparing the evoked responses in firing rate signals with the spontaneous activity in the prestimulus intervals at $\bar{g}_{\text{AMPA}} = 2$ and $\beta = 5$. The yellow horizontal dashed line defines the critical t-value. The concatenation of consecutive t-values above and below this threshold that forms the t-cluster statistics is shown as rectangles in the bottom line. Significant and non significant t-cluster statistics are indicated in red and black, respectively. The area and duration of the first t-cluster statistic, shown in gray, are used to quantify response propagation to the unperturbed population. Stimulus duration is represented by a black horizontal line. (B) Changes in the area of the first significant t-cluster statistic in seconds (y-axis) with respect to β (x-axis). (C) Changes in the length of the first significant t-cluster statistic in milliseconds (y-axis) with respect to β (x-axis). Violet, dark violet and indigo correspond to $\bar{g}_{\text{AMPA}} = 2$, $\bar{g}_{\text{AMPA}} = 6$ and $\bar{g}_{\text{AMPA}} = 10$, respectively. Figures show that the propagated information to the unperturbed population depends on β. Increasing $\bar{g}_{\text{AMPA}}$ from 2 to 10 and keeping $\beta = 1$ does not lead to a significant cluster in the unperturbed population (black squares). Increasing β from 1 to 3 shows a significant cluster (red squares) in all values of $\bar{g}_{\text{AMPA}}$. In the case of $\bar{g}_{\text{AMPA}} = 2$, the t-cluster statistics are significant ($P < 0.001$) for β =2, 3, 4 and 5. In the case of $\bar{g}_{\text{AMPA}} = 6$, the t-cluster statistics are significant ($P < 0.001$) for $\beta > 2$. In the case of $\bar{g}_{\text{AMPA}} = 10$, the t-cluster statistics are significant ($P < 0.001$) for $\beta > 2$.

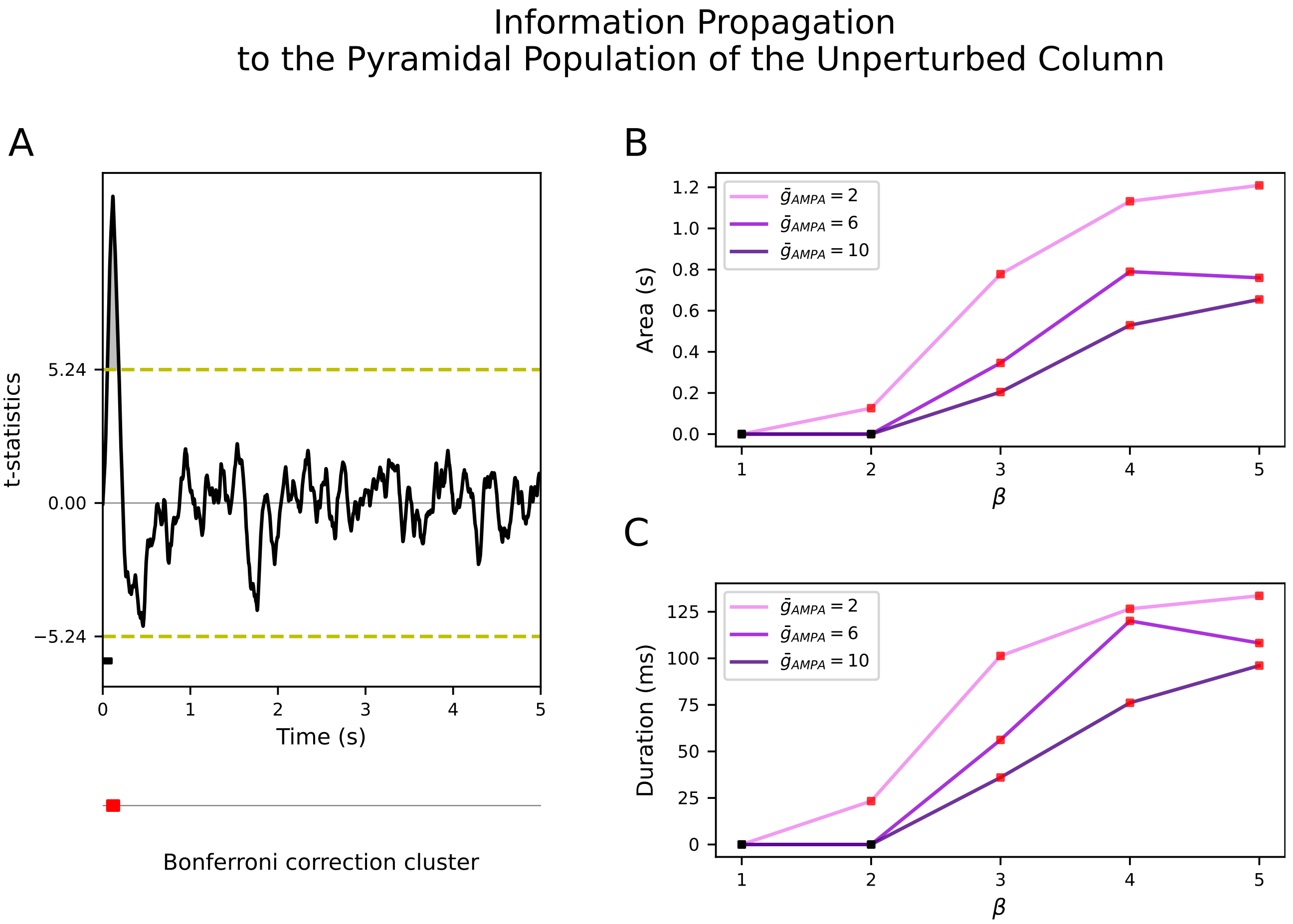

Figure S8. Information propagation as a function of β and $\bar{g}_{\mathrm{AMPA}}$ as in Figure 6 but for Bonferroni correction rather than temporal clustering nonparametric test. (A) Example of extraction of temporal cluster of Bonferroni correction when comparing the evoked responses in firing rate signals with the spontaneous activity in the prestimulus intervals at $\bar{g}_{\mathrm{AMPA}} = 2$ and $\beta = 5$. The yellow horizontal dashed line defines the Bonferroni modified critical t-value. The concatenation of consecutive t-values above and below this threshold that forms the temporal cluster of Bonferroni correction is shown as rectangles in the bottom line. The temporal cluster of Bonferroni correction is indicated in red. The area and duration of the first temporal cluster of Bonferroni correction, shown in gray, are used to quantify response propagation to the unperturbed population. Stimulus duration is represented by a black horizontal line. Note that the Bonferroni modified critical t-value is defined by is at the Bonferroni-corrected significance level of 0.01/50000 -where 50000 is the number of data points of multiple comparison- that corresponds to a critical t-value of 5.28. As shown, the bonferroni correction is less sensitive to significant deflections in the signal compared to the temporal clustering nonparametric test. Moreover, the Bonferroni correction method loses more its sensitivity to the significance detection as simulation time expands more. (B) Changes in the area of the first temporal cluster of Bonferroni correction in seconds (y-axis) with respect to β (x-axis). (C) Changes in the length of the first temporal cluster of Bonferroni correction in milliseconds (y-axis) with respect to β (x-axis). Violet, dark violet and indigo correspond to $\bar{g}_{\mathrm{AMPA}} = 2$, $\bar{g}_{\mathrm{AMPA}} = 6$ and $\bar{g}_{\mathrm{AMPA}} = 10$, respectively. Figures show that the propagated information to the unperturbed population depends on β. Increasing $\bar{g}_{\mathrm{AMPA}}$ from 2 to 10 and keeping $\beta = 1$ does not lead to a significant cluster in the unperturbed population (black squares). Increasing β from 1 to 3 shows a significant cluster (red squares) in all values of $\bar{g}_{\mathrm{AMPA}}$. In the case of $\bar{g}_{\mathrm{AMPA}} = 2$, there are temporal clusters of Bonferroni correction for $\beta > 1$. In the case of $\bar{g}_{\mathrm{AMPA}} = 6$, there are temporal clusters of Bonferroni correction for $\beta > 3$. In the case of $\bar{g}_{\mathrm{AMPA}} = 10$, there are temporal clusters of Bonferroni correction for $\beta > 3$.

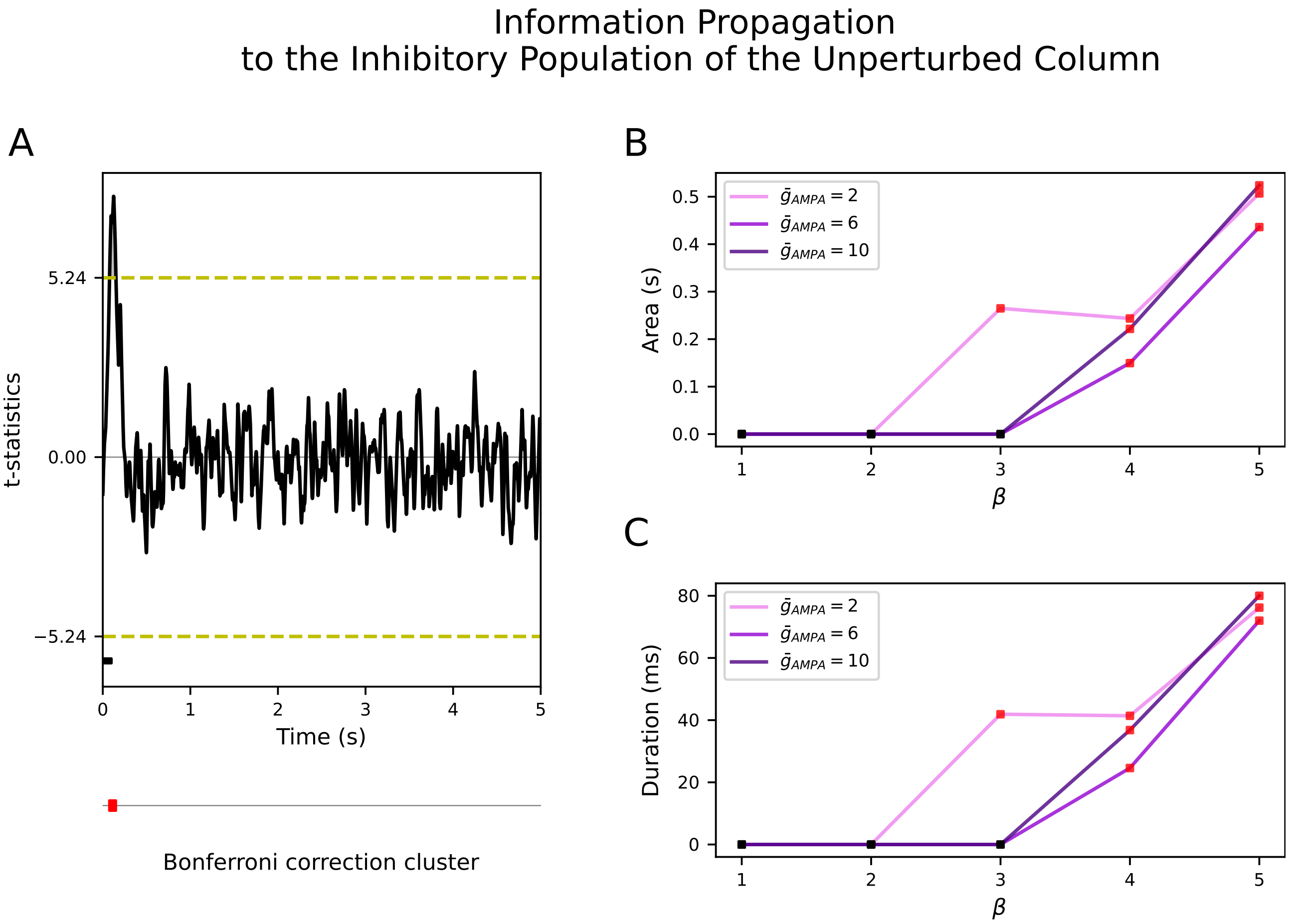

Figure S9. Information propagation as a function of β and $\bar{g}_{\text{AMPA}}$ as in Figure S8 but for inhibitory population. (A) Example of extraction of temporal cluster of Bonferroni correction when comparing the evoked responses in firing rate signals with the spontaneous activity in the prestimulus intervals at $\bar{g}_{\text{AMPA}} = 2$ and $\beta = 5$. The yellow horizontal dashed line defines the Bonferroni modified critical t-value. The concatenation of consecutive t-values above and below this threshold that forms the temporal cluster of Bonferroni correction is shown as rectangles in the bottom line. The temporal cluster of Bonferroni correction is indicated in red. The area and duration of the first temporal cluster of Bonferroni correction, shown in gray, are used to quantify response propagation to the unperturbed population. Stimulus duration is represented by a black horizontal line. (B) Changes in the area of the first temporal cluster of Bonferroni correction in seconds (y-axis) with respect to β (x-axis). (C) Changes in the length of the first temporal cluster of Bonferroni correction in milliseconds (y-axis) with respect to β (x-axis). Violet, dark violet and indigo correspond to $\bar{g}_{\text{AMPA}} = 2$, $\bar{g}_{\text{AMPA}} = 6$ and $\bar{g}_{\text{AMPA}} = 10$, respectively. Figures show that the propagated information to the unperturbed population depends on β. Increasing $\bar{g}_{\text{AMPA}}$ from 2 to 10 and keeping $\beta = 1$ does not lead to a significant cluster in the unperturbed population (black squares). Increasing β from 1 to 4 shows a significant cluster (red squares) in all values of $\bar{g}_{\text{AMPA}}$. In the case of $\bar{g}_{\text{AMPA}} = 2$, there are temporal clusters of Bonferroni correction for $\beta > 2$. In the case of $\bar{g}_{\text{AMPA}} = 6$, there are temporal clusters of Bonferroni correction for $\beta > 3$. In the case of $\bar{g}_{\text{AMPA}} = 10$, there are temporal clusters of Bonferroni correction for $\beta > 3$.

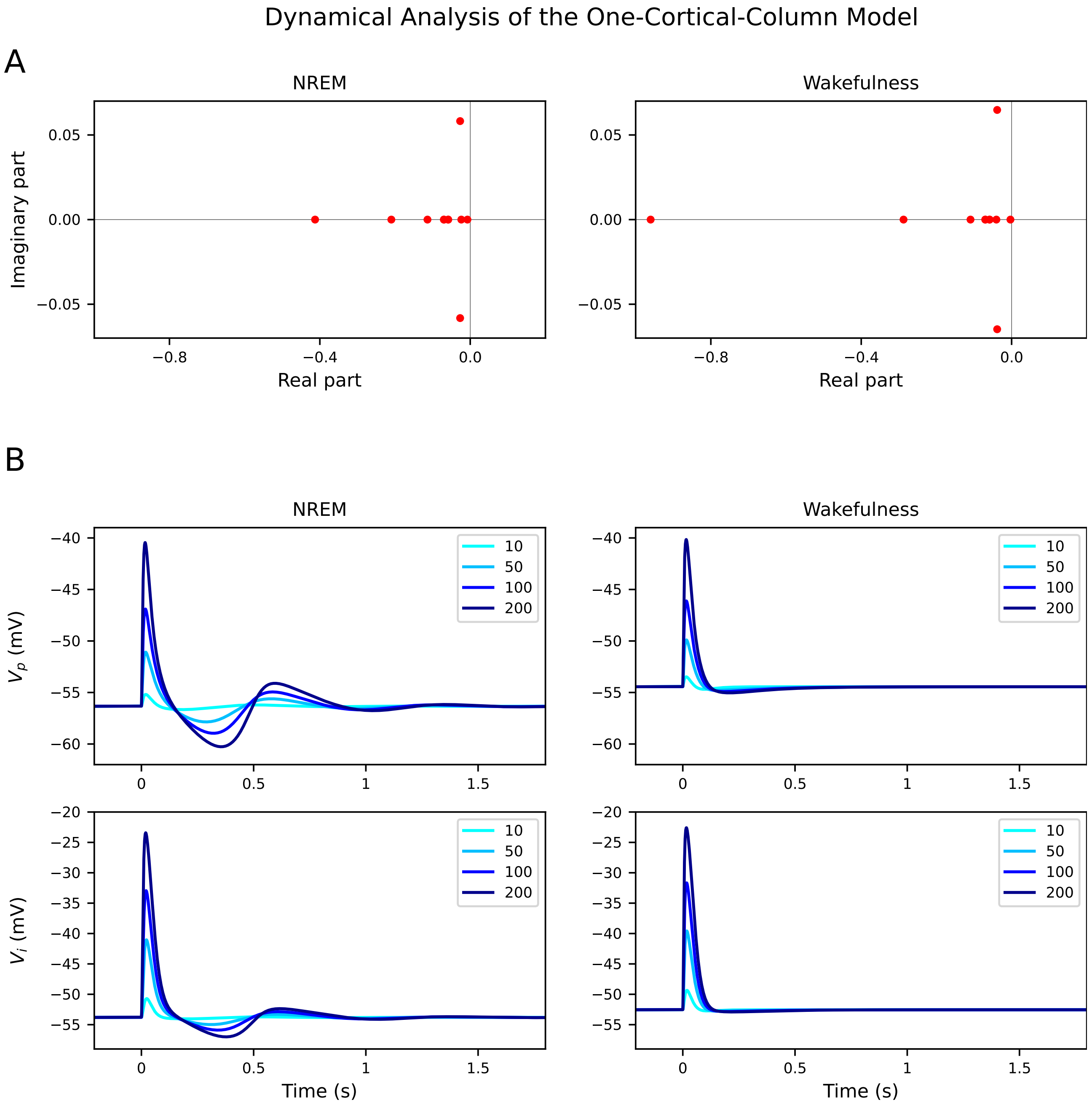

Figure S10. Dynamical analysis of one-cortical-column model. (A) Applying the linearization technique to the differential equations in Appendix A.2, shows that the real part of all eigenvalues is negative in NREM sleep (left column) and wakefulness (right column). Also, in both states there are two complex conjugate eigenvalues. This is indicative of the presence of a stable focus (also known as stable spiral). Note that there are 11 eigenvalues (red circles), corresponding to the number of variables in the system, although only 9 are visible due to the overlapping values between three of them. (B) Response of V_p (top row) and V_i (bottom row) in the absence of extrinsic noise ϕ to an instantaneous increase of the excitatory synapses. The amplitude of this increase is color coded from light to dark blue. The system produces damped oscillations in response to this perturbation at $t = 0$ for NREM sleep (left column) but not for wakefulness (right column). This behavior anticipates the emergence of slow oscillation in the presence of extrinsic noise in NREM sleep.
