# Supplementary material for: Computational Modeling of Information Propagation during the Sleep–Waking Cycle
Source: Biology (Basel). 2021 Sep 22;10(10):945. doi: 10.3390/biology10100945 (PMC8533346; doi:10.3390/biology10100945)

Evoked Response of Inhibitory Populations in the Two-Cortical-Column Model

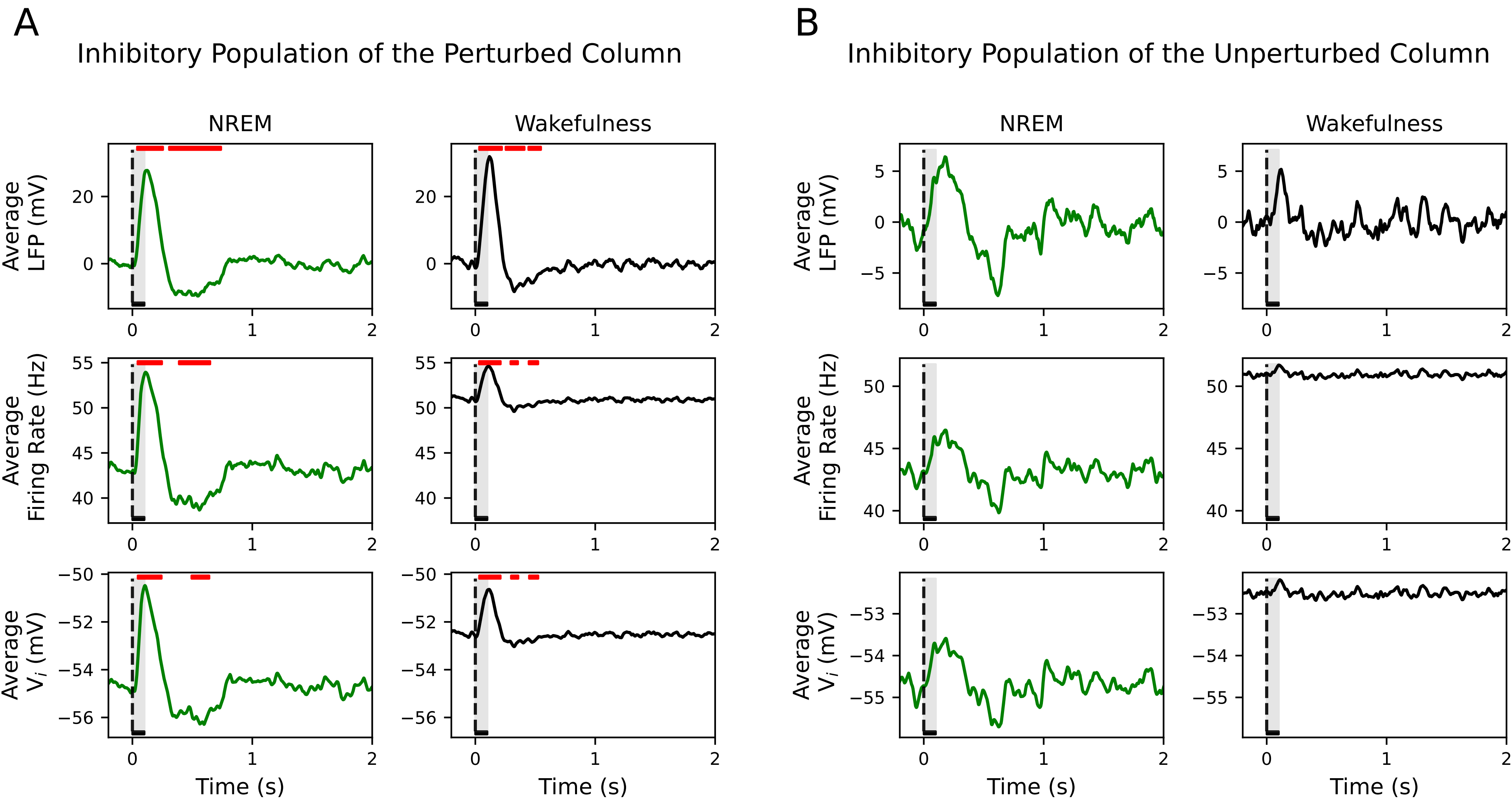

Supplement: Supplementary file 1 [file biology-10-00945-s001.zip › biology-1334040-supplementary/SUPP_FIGURES/POSTSTIMULUS_PLOT_TWO_CORTICAL_COLUMN_inh.pdf]
