# Supplementary material for: Computational Modeling of Information Propagation during the Sleep–Waking Cycle
Source: Biology (Basel). 2021 Sep 22;10(10):945. doi: 10.3390/biology10100945 (PMC8533346; doi:10.3390/biology10100945)
Supplement: Supplementary file 1 [file biology-10-00945-s001.zip › biology-1334040-supplementary/SUPP_FIGURES/POSTSTIMULUS_PLOT_UNPERTURBED_CORTICAL_COLUMN_SCREENING_Bonferroni_inh.pdf]

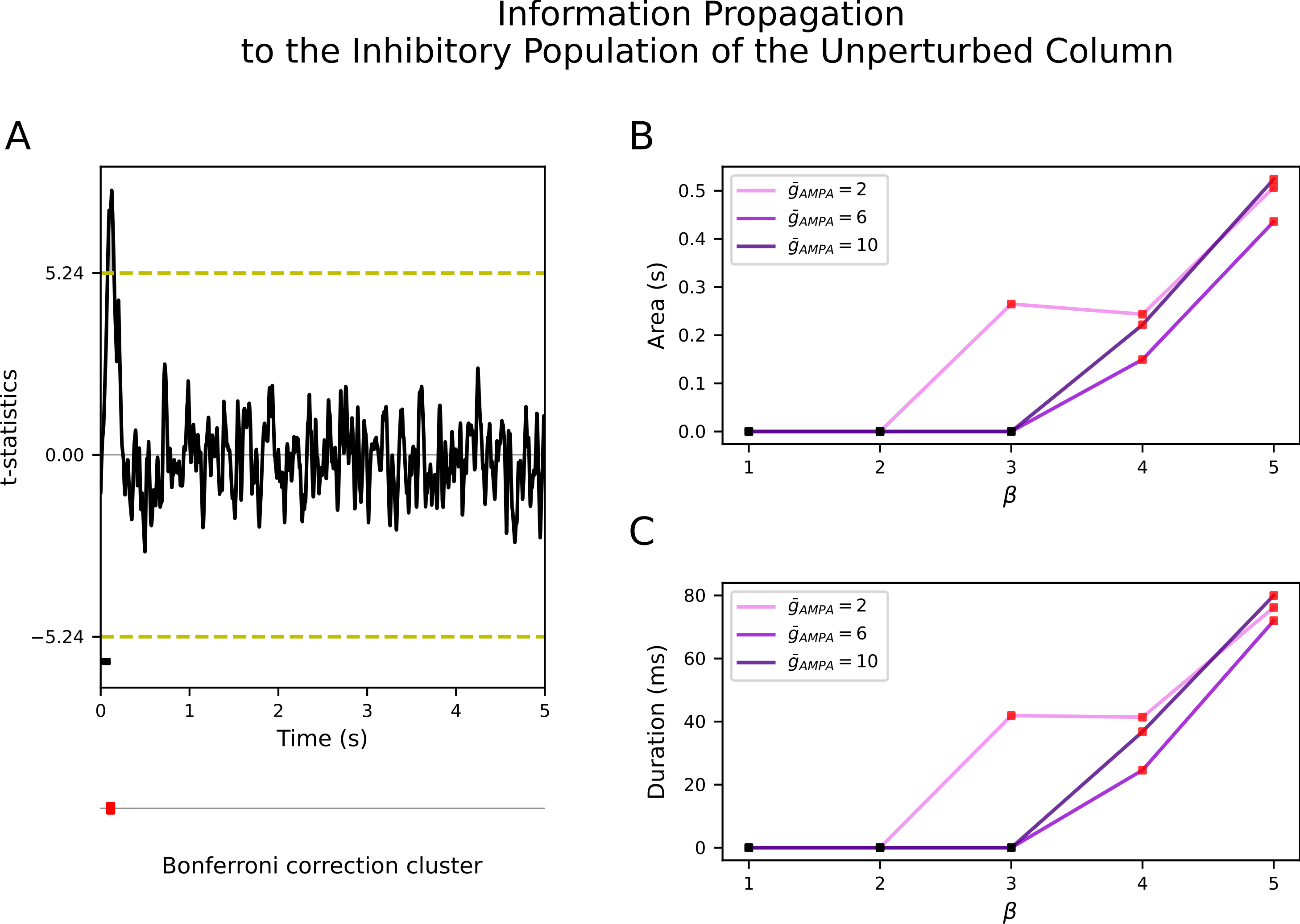
Information Propagation
to the Inhibitory Population of the Unperturbed Column
A
B
C
5.24
0.00
−5.24
t-statistics
0
1
2
3
4
5
Time (s)
Bonferroni correction cluster
$\bar{g}_{AMPA} = 2$
$\bar{g}_{AMPA} = 6$
$\bar{g}_{AMPA} = 10$
Area (s)
0.0
0.1
0.2
0.3
0.4
0.5
β
Duration (ms)
0
20
40
60
80
