# Supplementary material for: Computational Modeling of Information Propagation during the Sleep–Waking Cycle
Source: Biology (Basel). 2021 Sep 22;10(10):945. doi: 10.3390/biology10100945 (PMC8533346; doi:10.3390/biology10100945)
Supplement: Supplementary file 1 [file biology-10-00945-s001.zip › biology-1334040-supplementary/SUPP_FIGURES/POSTSTIMULUS_PLOT_UNPERTURBED_CORTICAL_COLUMN_SCREENING_Bonferroni_pyr.pdf]

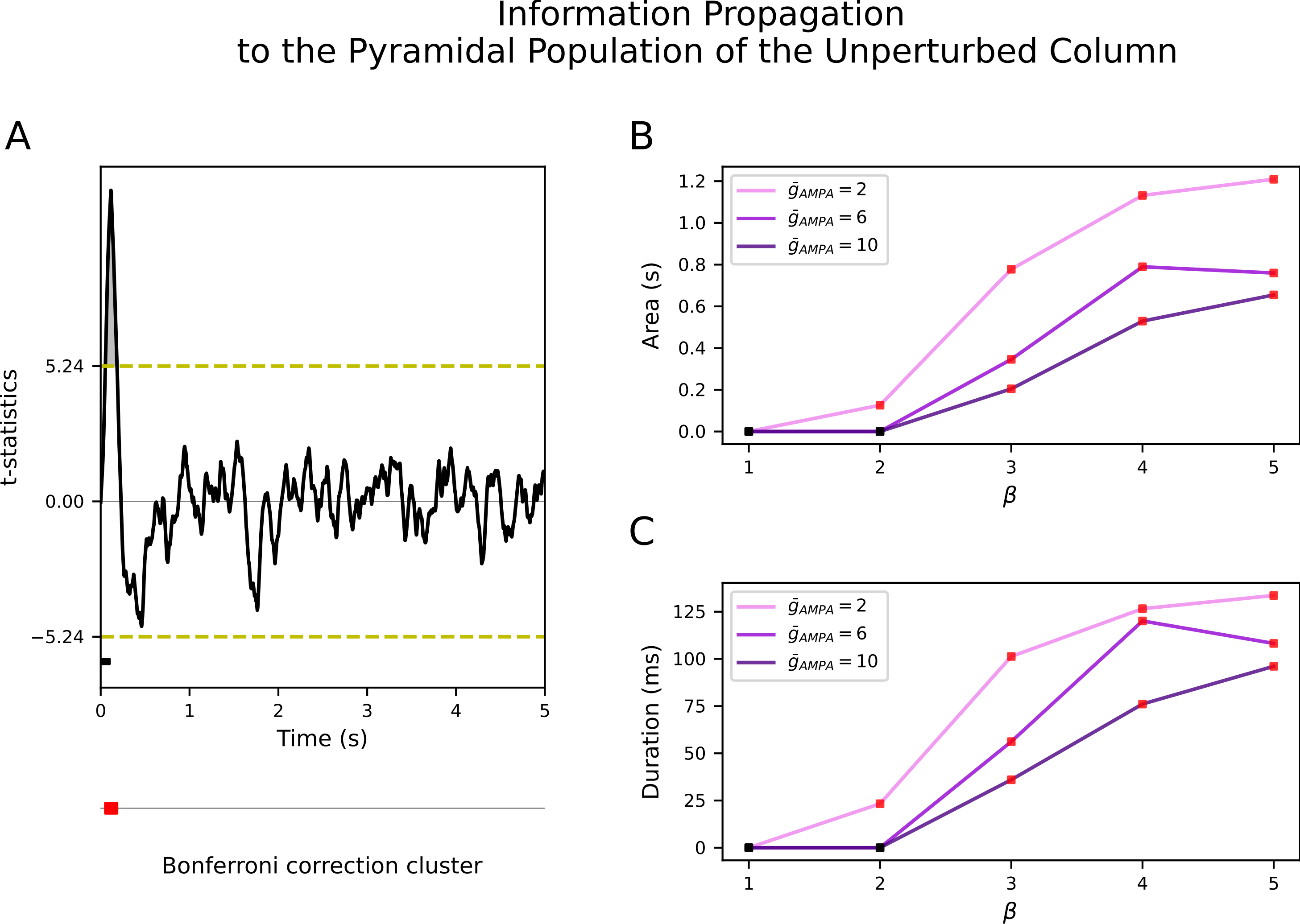
Information Propagation
to the Pyramidal Population of the Unperturbed Column
A
t-statistics
5.24
0.00
−5.24
0
1
2
3
4
5
Time (s)
Bonferroni correction cluster
B
Area (s)
1.2
1.0
0.8
0.6
0.4
0.2
0.0
$\bar{g}_{AMPA} = 2$
$\bar{g}_{AMPA} = 6$
$\bar{g}_{AMPA} = 10$
1
2
3
4
5
β
C
Duration (ms)
125
100
75
50
25
0
$\bar{g}_{AMPA} = 2$
$\bar{g}_{AMPA} = 6$
$\bar{g}_{AMPA} = 10$
1
2
3
4
5
β
