# Supplementary figures and images for: Computational Modeling of Information Propagation during the Sleep–Waking Cycle
Source: Biology (Basel). 2021 Sep 22;10(10):945. doi: 10.3390/biology10100945 (PMC8533346; doi:10.3390/biology10100945)

# Dynamical Analysis of the One-Cortical-Column Model

A

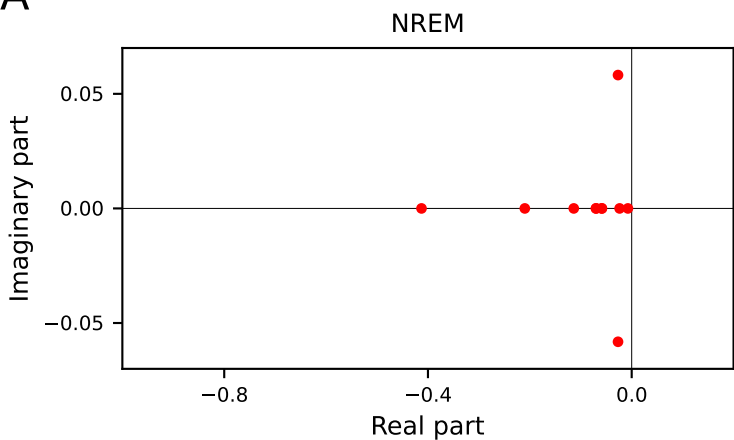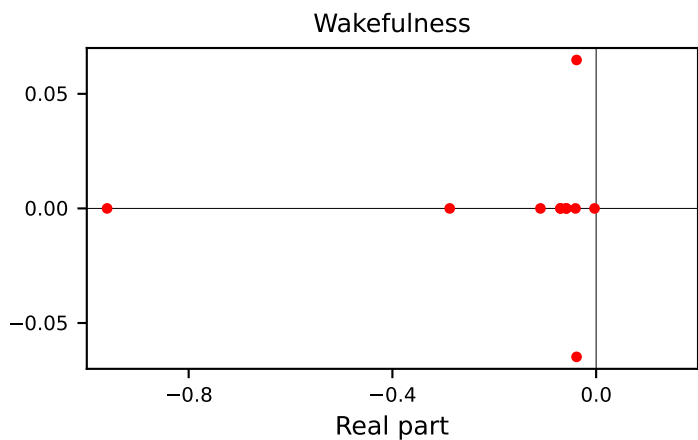

B

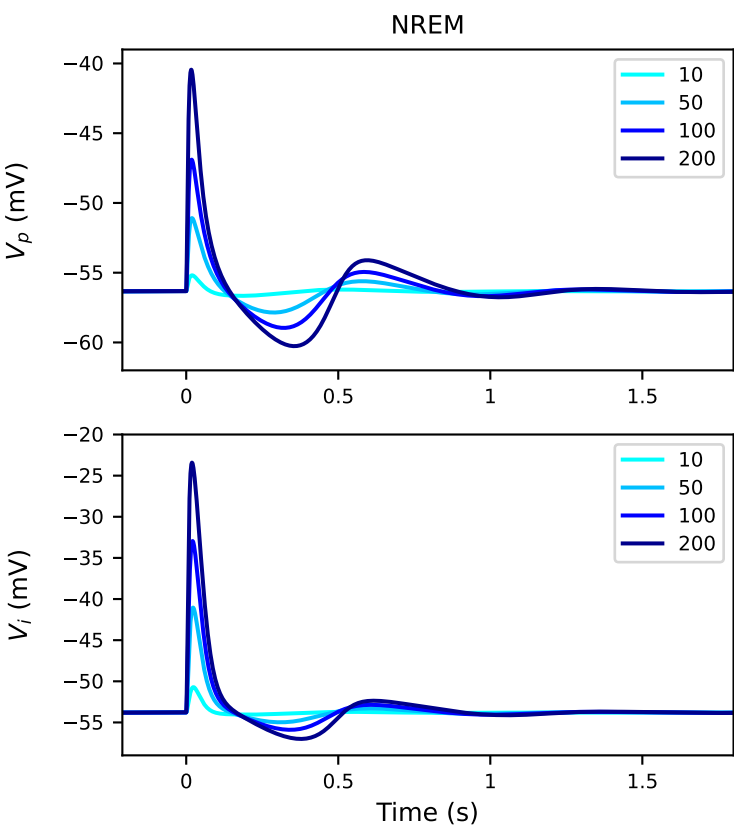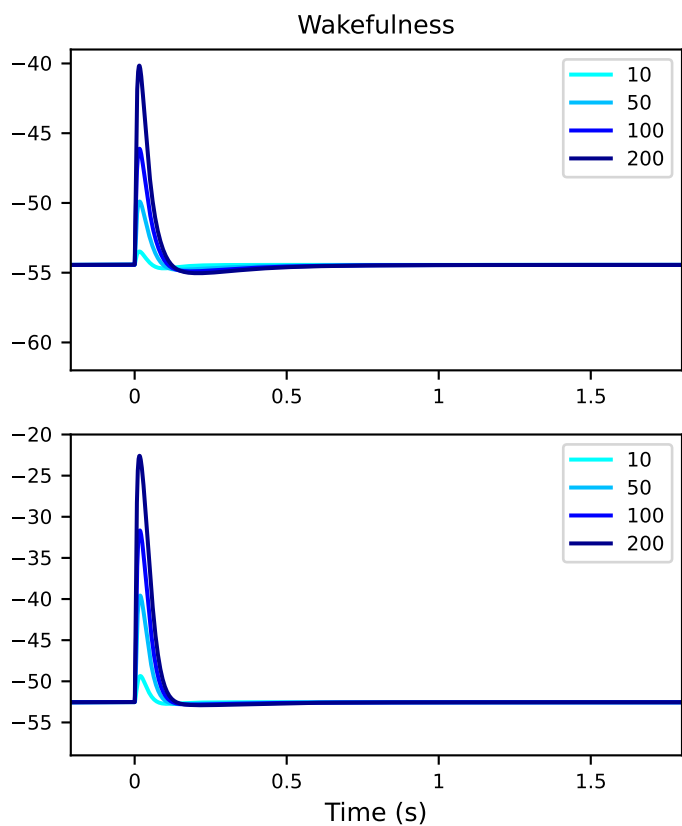

Supplement: Supplementary file 1 [file biology-10-00945-s001.zip › biology-1334040-supplementary/SUPP_FIGURES/DYNAMICAL_ANALYSIS.pdf]

# Evoked Response of Inhibitory Population in the One-Cortical-Column Model

**A**

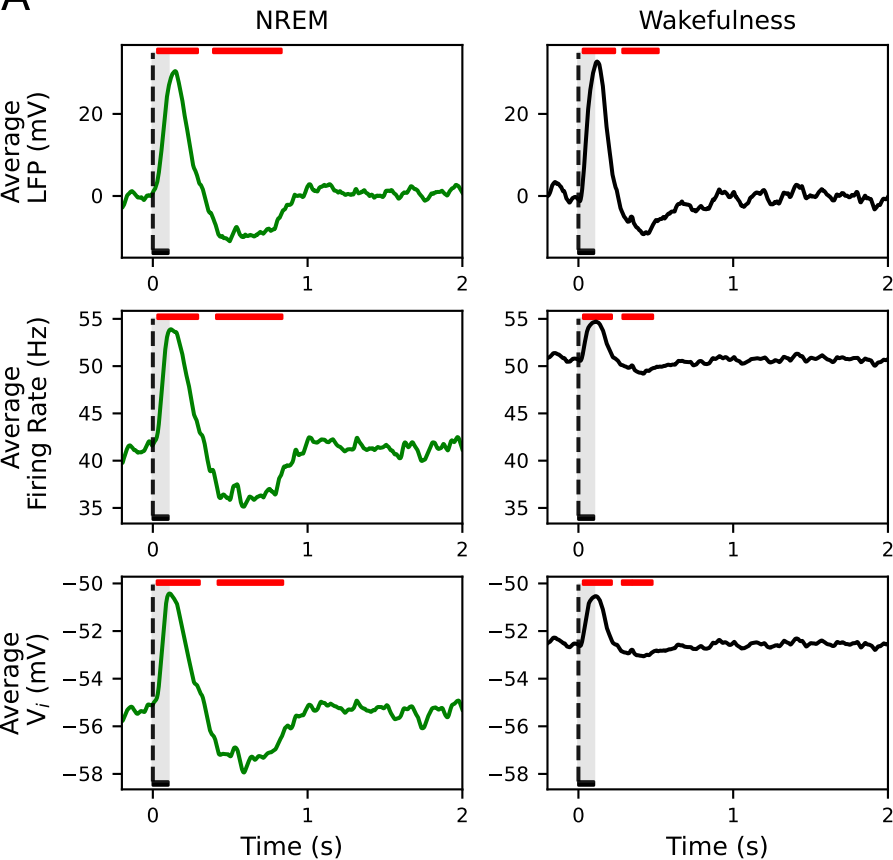

**B**

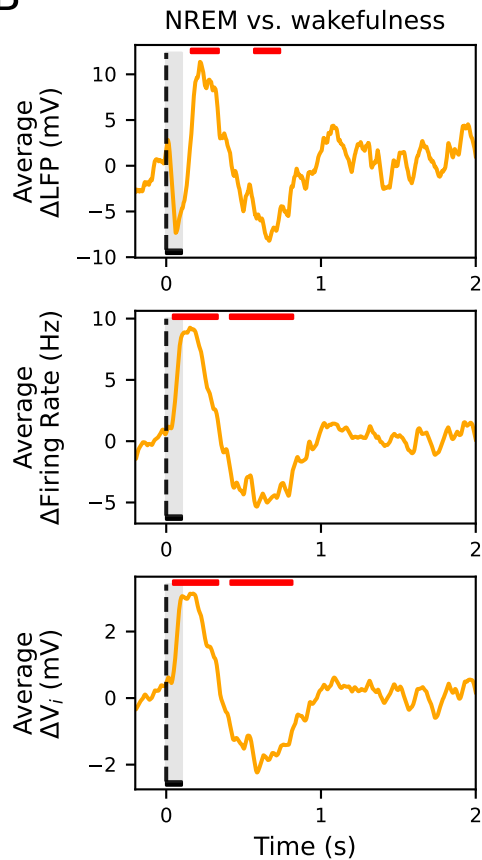

Supplement: Supplementary file 1 [file biology-10-00945-s001.zip › biology-1334040-supplementary/SUPP_FIGURES/POSTSTIMULUS_PLOT_ONE_CORTICAL_COLUMN_inh.pdf]

# Inhibitory Population of the Unperturbed Column

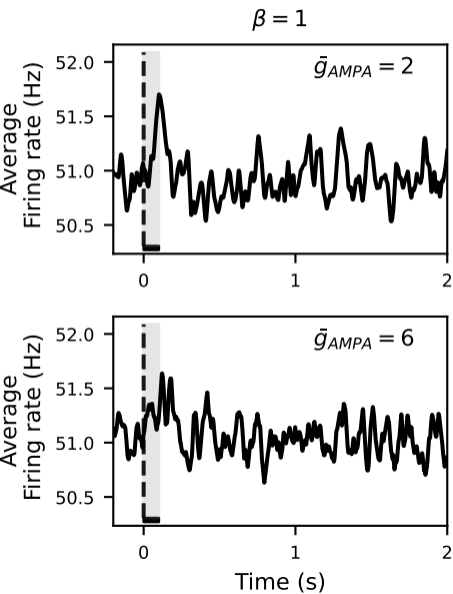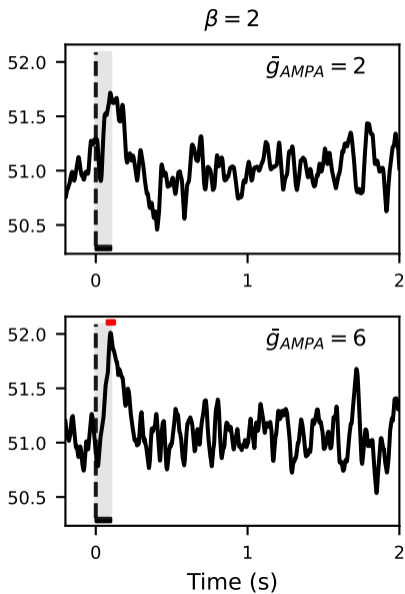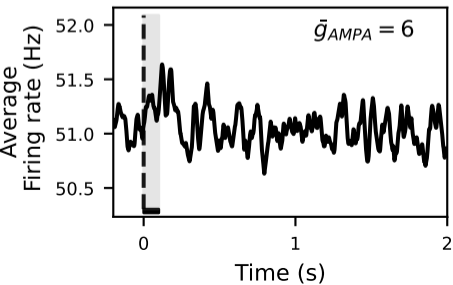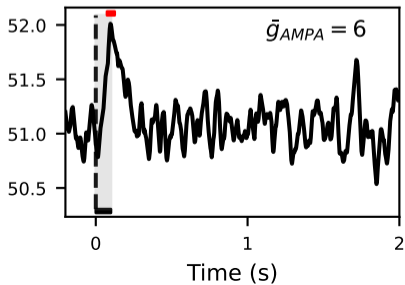

Supplement: Supplementary file 1 [file biology-10-00945-s001.zip › biology-1334040-supplementary/SUPP_FIGURES/POSTSTIMULUS_PLOT_UNPERTURBED_CORTICAL_COLUMN_inh.pdf]

# Information Flow to the Inhibitory Population of the Unperturbed Column

A

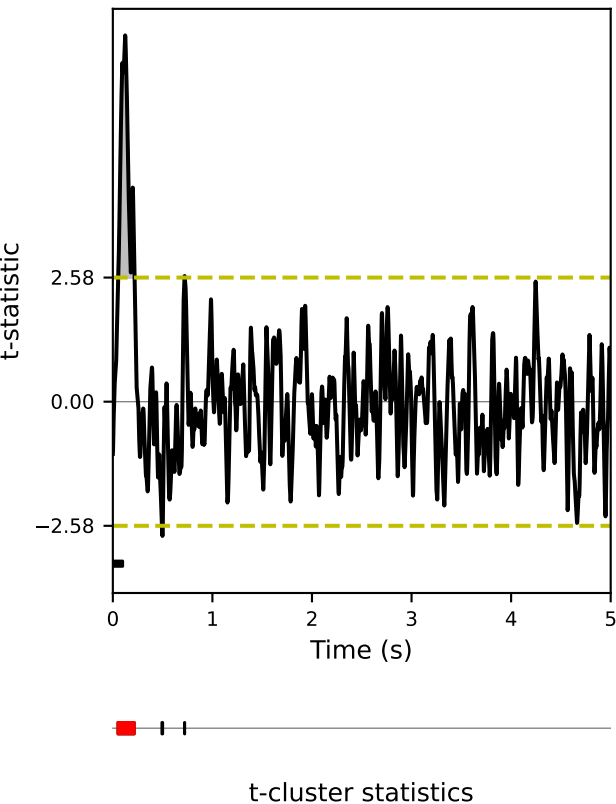

B

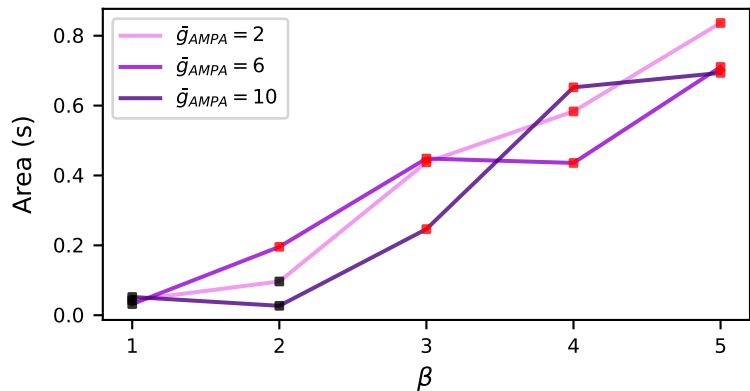

C

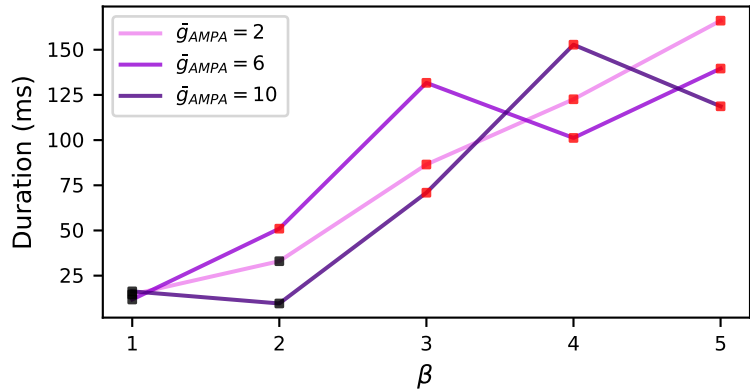

Supplement: Supplementary file 1 [file biology-10-00945-s001.zip › biology-1334040-supplementary/SUPP_FIGURES/POSTSTIMULUS_PLOT_UNPERTURBED_CORTICAL_COLUMN_SCREENING_inh.pdf]

# Spontaneous Activity of Inhibitory Population in the One-Cortical-Column Model

**A**

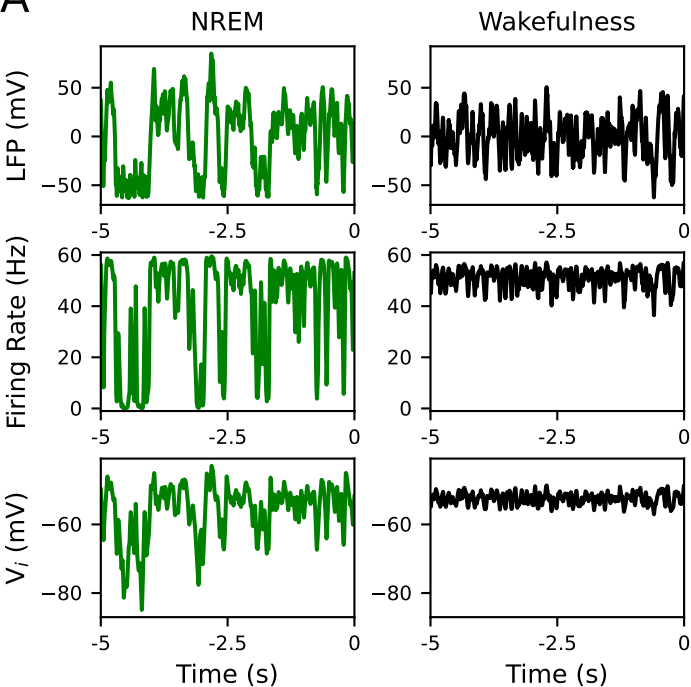

**B**

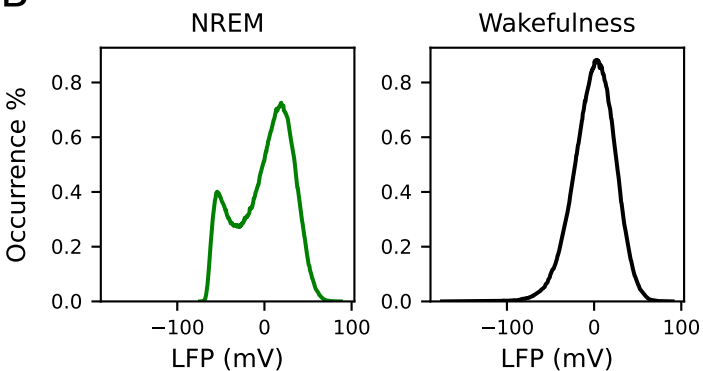

**C**

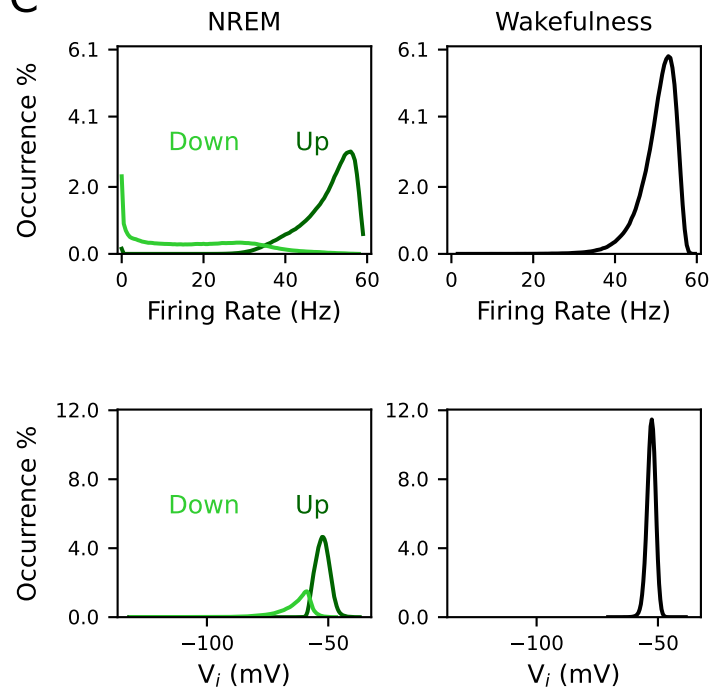

**D**

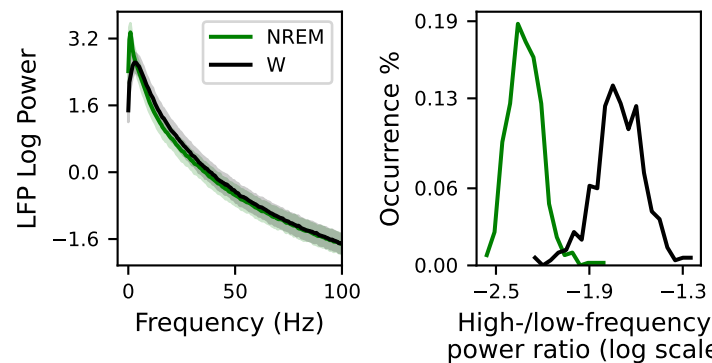

Supplement: Supplementary file 1 [file biology-10-00945-s001.zip › biology-1334040-supplementary/SUPP_FIGURES/PRESTIMULUS_PLOT_ONE_CORTICAL_COLUMN_Perturbed_inh.pdf]

# Spontaneous Activity of Inhibitory Population in the Two-Cortical-Column Model

**A**

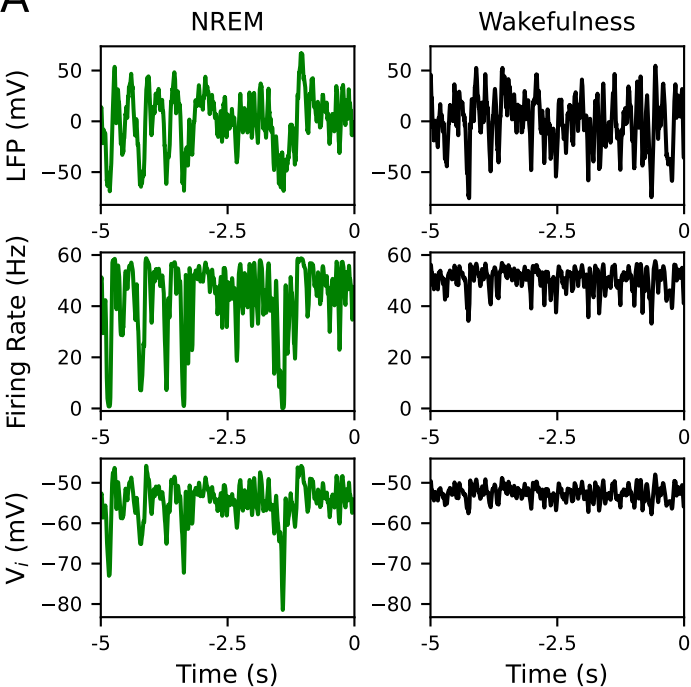

**B**

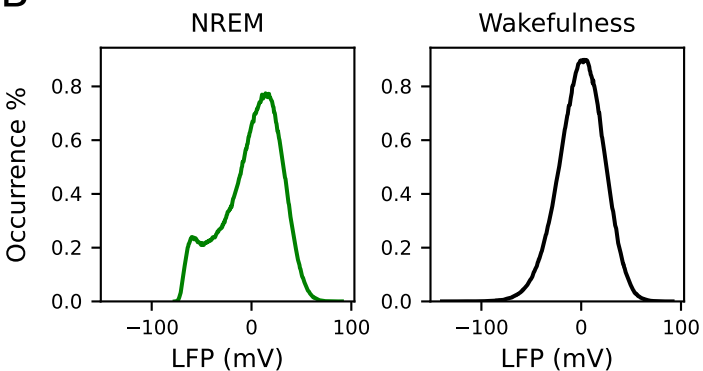

**C**

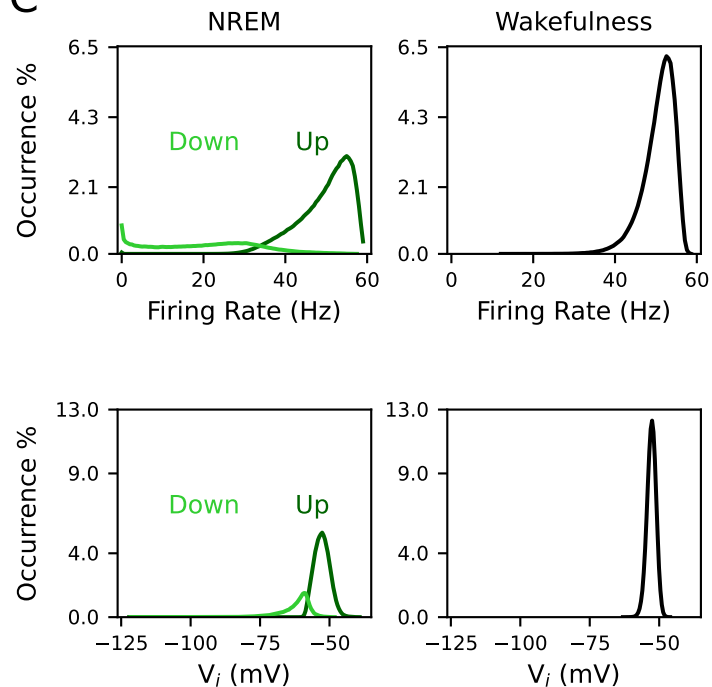

**D**

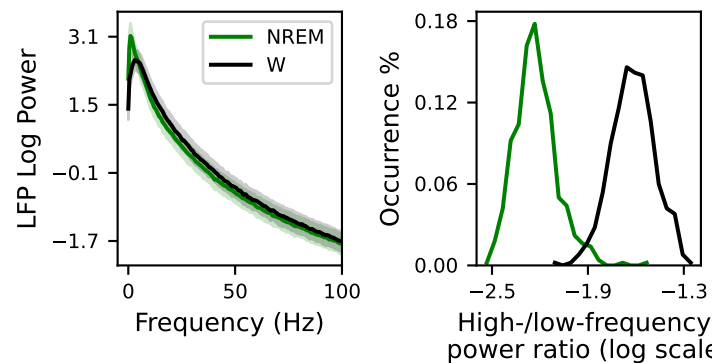

Supplement: Supplementary file 1 [file biology-10-00945-s001.zip › biology-1334040-supplementary/SUPP_FIGURES/PRESTIMULUS_PLOT_TWO_CORTICAL_COLUMN_Perturbed_inh.pdf]

# Spontaneous Activity of Pyramidal Population in the Two-Cortical-Column Model

**A**

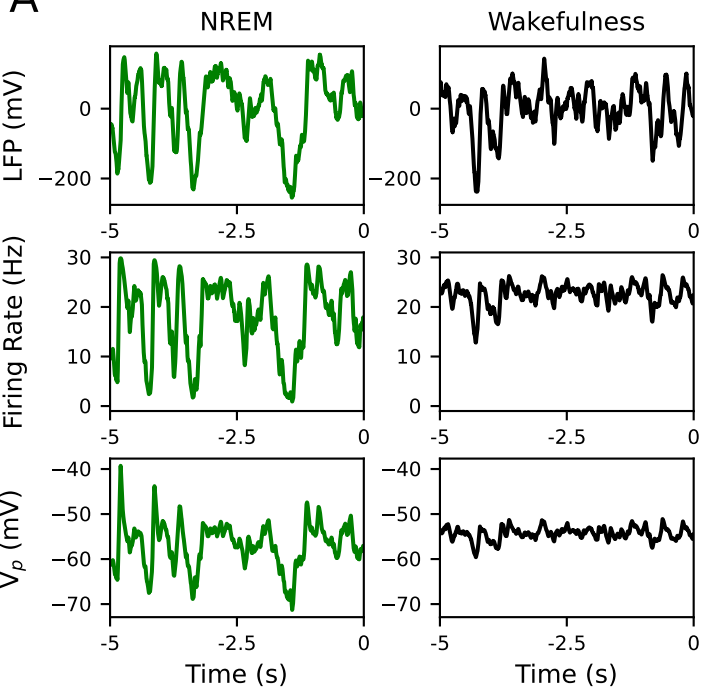

**B**

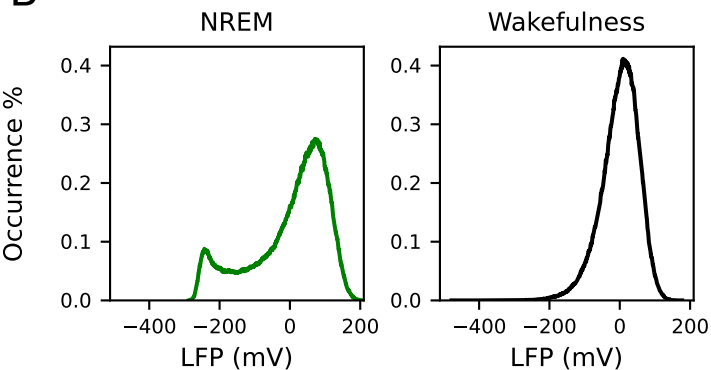

**C**

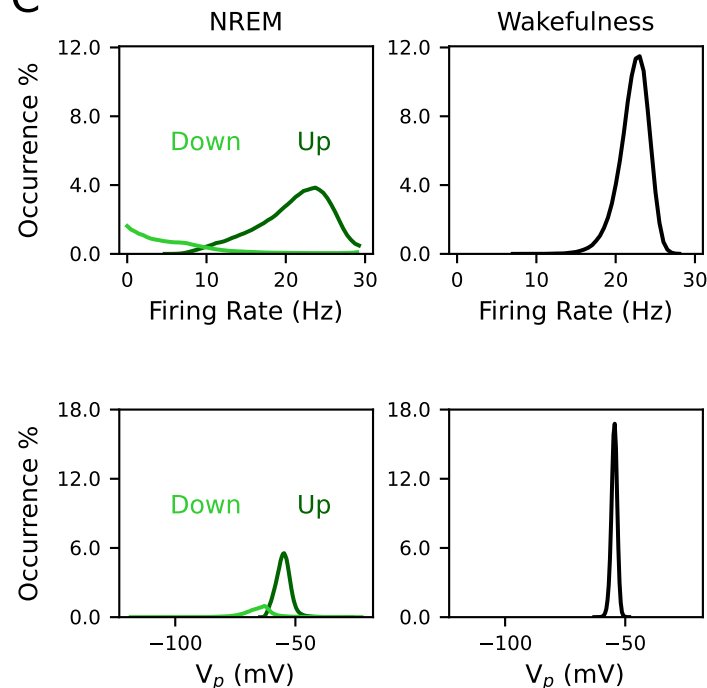

**D**

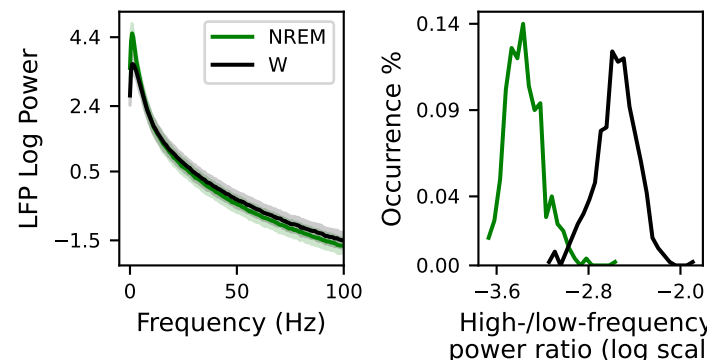

Supplement: Supplementary file 1 [file biology-10-00945-s001.zip › biology-1334040-supplementary/SUPP_FIGURES/PRESTIMULUS_PLOT_TWO_CORTICAL_COLUMN_Perturbed_pyr.pdf]

# Spontaneous Activity of Inhibitory Population in the Two-Cortical-Column Model

**A**

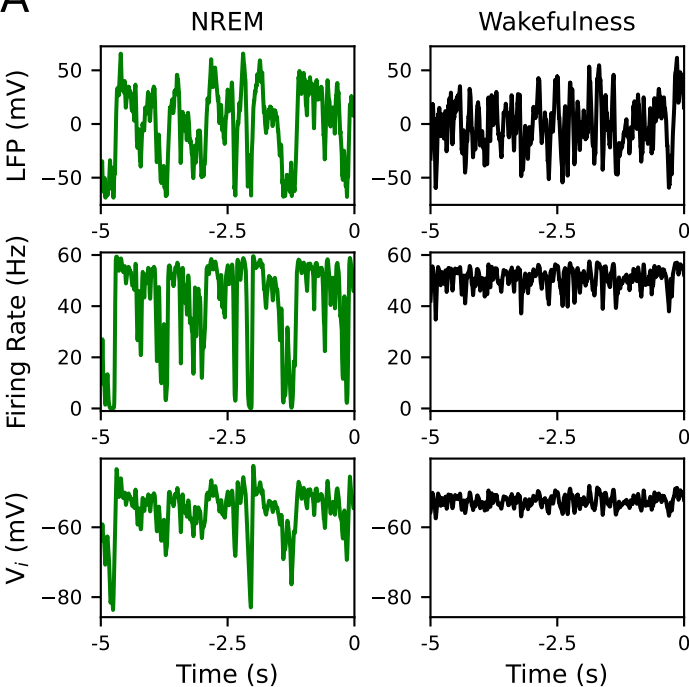

**B**

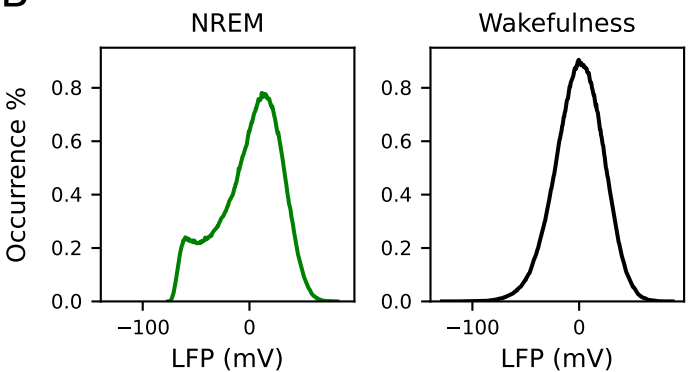

**C**

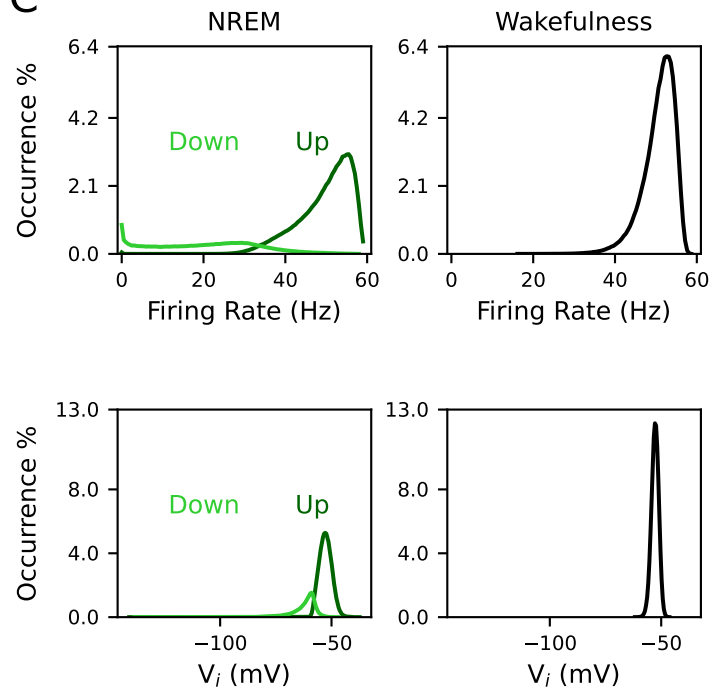

**D**

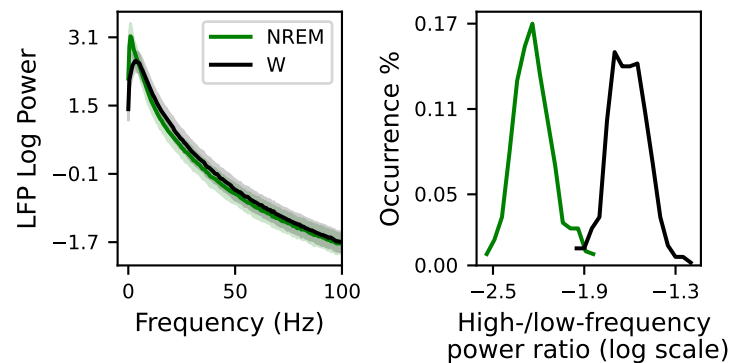

Supplement: Supplementary file 1 [file biology-10-00945-s001.zip › biology-1334040-supplementary/SUPP_FIGURES/PRESTIMULUS_PLOT_TWO_CORTICAL_COLUMN_Unperturbed_inh.pdf]

# Spontaneous Activity of Pyramidal Population in the Two-Cortical-Column Model

**A**

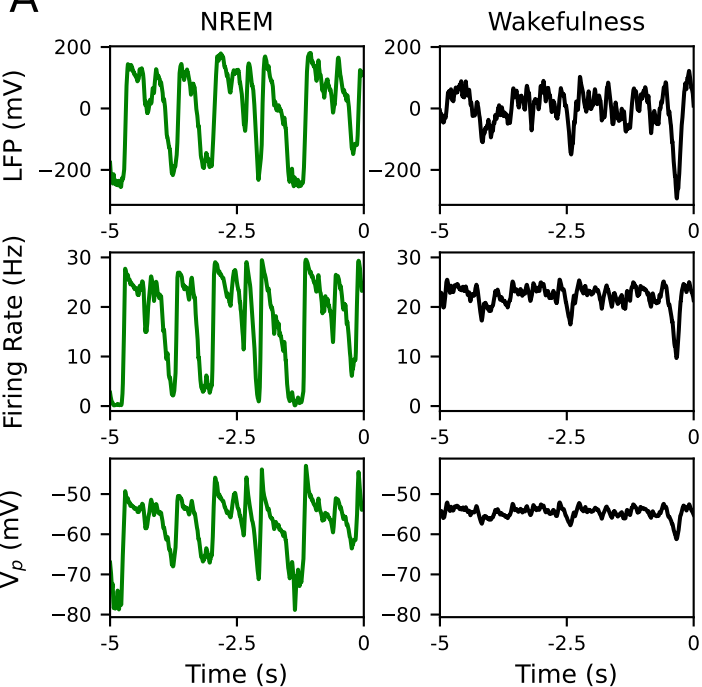

**B**

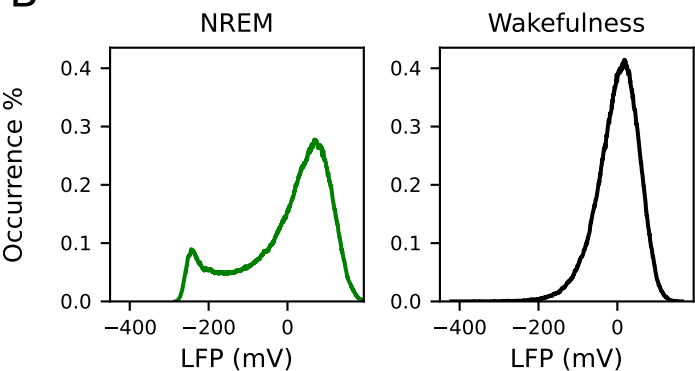

**C**

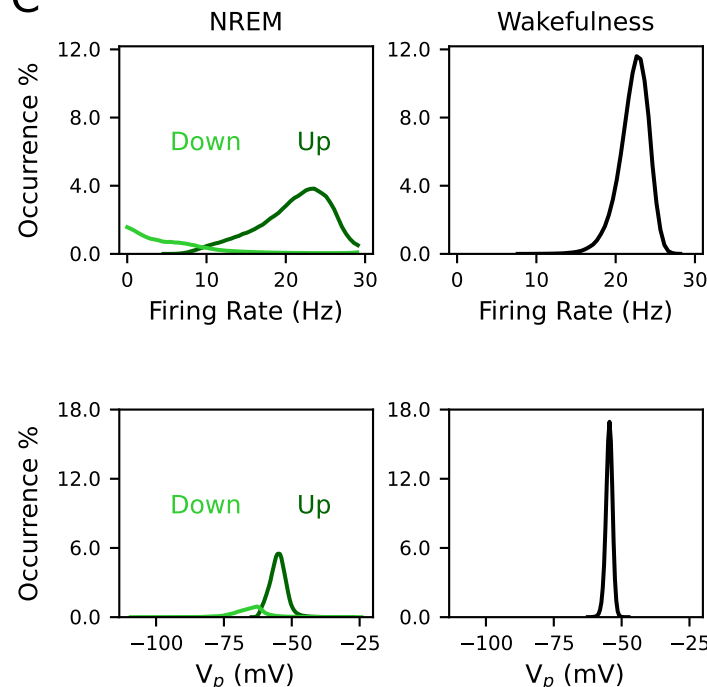

**D**

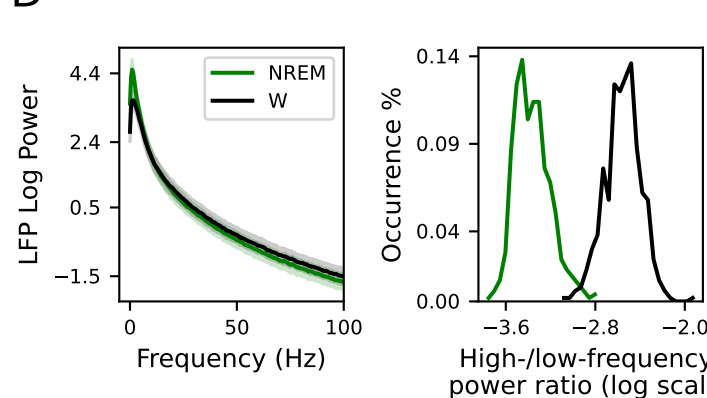

Supplement: Supplementary file 1 [file biology-10-00945-s001.zip › biology-1334040-supplementary/SUPP_FIGURES/PRESTIMULUS_PLOT_TWO_CORTICAL_COLUMN_Unperturbed_pyr.pdf]

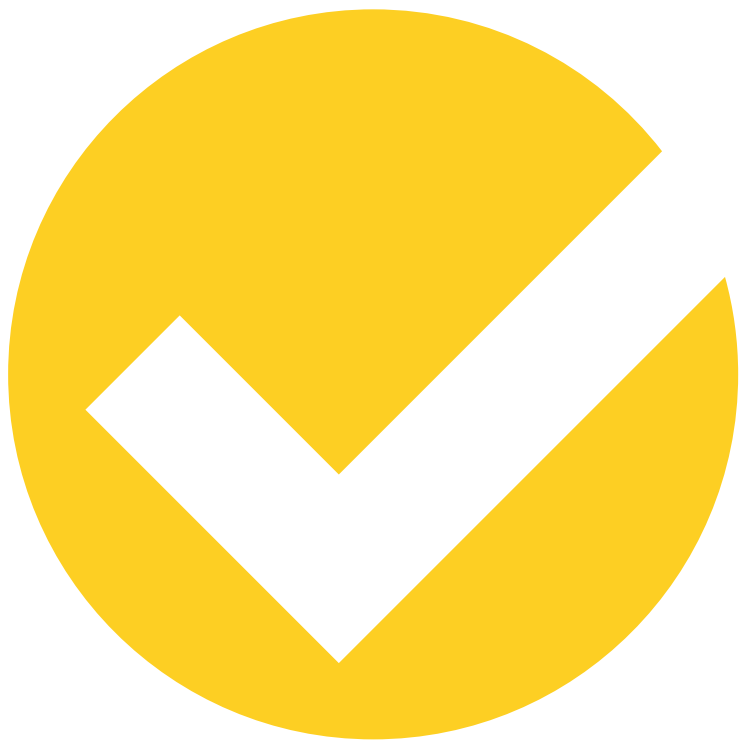

check for  
updates

Supplement: Supplementary file 1 [file biology-10-00945-s001.zip › biology-1334040-supplementary/Definitions/logo-updates.pdf]

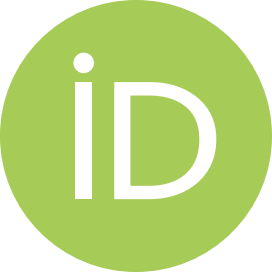

Supplement: Supplementary file 1 [file biology-10-00945-s001.zip › biology-1334040-supplementary/Definitions/logo-orcid-eps-converted-to.pdf]
